# Mouse models of human ocular disease for translational research

**DOI:** 10.1371/journal.pone.0183837

**Published:** 2017-08-31

**Authors:** Mark P. Krebs, Gayle B. Collin, Wanda L. Hicks, Minzhong Yu, Jeremy R. Charette, Lan Ying Shi, Jieping Wang, Jürgen K. Naggert, Neal S. Peachey, Patsy M. Nishina

**Affiliations:** 1 The Jackson Laboratory, Bar Harbor, Maine, United States of America; 2 Department of Ophthalmic Research, Cole Eye Institute, Cleveland Clinic Foundation, Cleveland, Ohio, United States of America; 3 Department of Ophthalmology, Cleveland Clinic Lerner College of Medicine of Case Western Reserve University, Cleveland, Ohio, United States of America; 4 Research Service, Louis Stokes Cleveland VA Medical Center, Cleveland, Ohio, United States of America; Dalhousie University, CANADA

## Abstract

Mouse models provide a valuable tool for exploring pathogenic mechanisms underlying inherited human disease. Here, we describe seven mouse models identified through the Translational Vision Research Models (TVRM) program, each carrying a new allele of a gene previously linked to retinal developmental and/or degenerative disease. The mutations include four alleles of three genes linked to human nonsyndromic ocular diseases (*Aipl1*^*tvrm119*^, *Aipl1*^*tvrm127*^, *Rpgrip1*^*tvrm111*^, *Rho*^*Tvrm334*^) and three alleles of genes associated with human syndromic diseases that exhibit ocular phentoypes (*Alms1*^*tvrm102*^, *Clcn2*^*nmf289*^, *Fkrp*^*tvrm53*^). Phenotypic characterization of each model is provided in the context of existing literature, in some cases refining our current understanding of specific disease attributes. These murine models, on fixed genetic backgrounds, are available for distribution upon request and may be useful for understanding the function of the gene in the retina, the pathological mechanisms induced by its disruption, and for testing experimental approaches to treat the corresponding human ocular diseases.

## Introduction

The laboratory mouse is the most widely used mammalian model for biomedical research. Classical genetic and targeted gene engineering approaches have yielded a large number of mouse models that replicate key features of human genetic conditions, including eye disease. With proper attention to strain genetic background and control of experimental variables, such as environmental conditions and diet, these models can provide important insights into disease mechanisms, and be used as a resource for preclinical studies and development of therapeutic interventions.

However, despite the availability of a large number of mouse models, some limitations exist in their use for modeling human diseases. First, the vast majority of models are null mutants, reflecting the preference to target early exons by homologous recombination [1]. By contrast, the mutations underlying human genetic diseases typically are missense mutations that involve a mechanism other than a complete loss of function, such as constitutive activation [2–4], altered ligand binding [3] or reduced activity [5]. Second, for many disease genes, only one or a small number of mouse models with different alleles have been developed. Therefore, distinct genetic variants that can number into the hundreds in humans, many with different clinical consequences, are not available [6]. In addition, targeted mutant models created by homologous recombination are often on mixed genetic backgrounds, which may result in unexpected phenotypes that confound the comparison of allelic differences. For example, in a traditional strategy, mice produced from homologous gene targeting in C57BL/6N (B6N)-derived embryonic stem cells are crossed to C57BL/6J (B6J) mice. In several notable cases, this practice produced ocular phenotypes that were independent of the targeted mutation(s) and instead traced to the introduction of the *Crb1*^*rd8*^ mutation [7], [8], which is present in B6N-derived mice [7], [9], [10]. This mutation causes a retinal degenerative phenotype with dysplastic lesions that vary with strain genetic background, complicating the interpretation of targeted mutant phenotypes [7], [11], [12]. Thus, a constant and well-characterized genetic background is critical for comparing phenotypic features and therapeutic responses among disease-associated alleles.

Chemical mutagenesis programs have been established worldwide to identify large numbers of mouse mutants for research in a broad range of biological systems (mutagenetix.utsouthwestern.edu, australianphenomics.org.au, www.mouseclinic.de, www.har.mrc.ac.uk/research/large-scale-functional-genomics/eumodic) [13–23]. For studying diseases of the visual system, the Translational Vision Research Models (TVRM) program at The Jackson Laboratory (JAX) uses ocular screens to identify genetic models created through ENU mutagenesis of mice [24], [25]. The visual system is particularly amenable to non-invasive high-throughput screening that includes both morphological (for example, indirect ophthalmoscopy) and functional (for example, electroretinography) phenotyping approaches [26–29]. Here, we describe newly characterized models generated through the TVRM program that provide allelic series for human disease genes known to play important roles in retinal development, function or maintenance. The models generated from the TVRM program are either derived on or are incipient congenics of the B6J or BALB/cByJ backgrounds, without known mutations, such as *Crb1*^*rd8*^, *Pde6b*^*rd1*^ or *Gnat2*^*cpfl3*^, that result in retinal pathology [7], [9]. The models are free of exogenous sequences, such as drug-resistance cassettes or reporter genes, which can sometimes complicate the interpretation of mutant phenotypes. These new mouse mutants are available and will be useful in expanding knowledge of molecular disease mechanisms and therapeutic strategies for human ocular disease intervention.

## Results and discussion

In the TVRM program [24], [25], a total of 7,591 G_1_, G_2_, and G_3_ mice have been screened by indirect ophthalmoscopy and 5,770 of these have been examined by electroretinography (ERG) to assess cone photoreceptor responses. Ninety heritable ENU-induced ocular mutants have thus far been identified; 34 of these are available for distribution and their causative genetic mutations are described (S1 Table). The seven new mutant strains detailed in this report are classified according to the human disease most closely represented by the mutant phenotype (Table 1), and include four models of non-syndromic and three models of syndromic disease that have retinal dystrophic or degenerative manifestations. The strains carry allelic variants of genes that are known to confer an ocular phenotype when mutated and some exhibit distinct features that differ from previously reported alleles, suggesting potential domain effects. Thus, these mice provide or extend allelic series for the genes involved (see Table 1). In the following sections, we present an overview of ocular and systemic phenotypes of the new mutant lines with a description of how they compare to currently available mutants and how they may be useful to the vision research community.

**Table 1:** Ocular diseases and corresponding mouse models.

Disease^a^ (OMIM Designation)	Mutation^b^	Allele Type; Mutation/Effect^c^	Strain Background	Reference
Nonsyndromic				
LCA4, MIM# 604393	*Aipl1*^*tvrm119*^	Chemically induced (ENU); c.276+5G>A	B6J	this report
	*Aipl1*^*tvrm127*^	Chemically induced (ENU); p.Cys89Phe	B6J	this report rrrrepaaaaareport
	*Aipl1*^*tm1Mad*^	Targeted; insertion, intragenic deletion	129S7/SvEvBrd-*Hprt*^*b-m2*^	[30]
	*Aipl1*^*tm1Tili*^	Targeted; neomycin cassette insertion	129S4/SvJae, C57BL/6^d^	[31]
	*Aipl1*^*tm1Visu*^	Targeted; insertion, intragenic deletion	129S1/Sv, 129X1/SvJ, B6J	[32]
LCA6, MIM# 613826	*Rpgrip1*^*tvrm111*^	Chemically induced (ENU); c.813+1G>A	B6J	this report
	*Rpgrip1*^*nmf247*^	Chemically induced (ENU); c.683-1A>T	B6J	[33]
	*Rpgrip1*^*tm1Tili*^	Targeted; insertion	129S4/SvJae, C57BL/6^d^	[34]
RP4, MIM# 613731	*Rho*^*Tvrm334*^	Chemically induced (ENU); p.Tyr178Cys	B6J	this report
	*Rho*^*Noerg1*^	Chemically induced (ENU); p.Cys110Tyr	B6J	[35]
	*Rho*^*R3*^	Chemically induced (ENU); p.Cys185Arg	B6J	[36]
	*Rho*^*tm1*.1(RHO*)Akgr^	Targeted; p. Ter349Gluext*51	129S7/SvEvBrd-*Hprt*^*b-m2*^, BALB/c, FVB/N	[37]
	*Rho*^*tm1*.*1Eye*^	Targeted; p.Asp190Asn	129S6/SvEvTac, C57BL/6^d^	[38]
	*Rho*^*tm1*.*1KpaI*^	Targeted; p.Pro23His	129S6/SvEvTac, C57BL/6^d^, FVB/N	[39]
	*Rho*^*tm1Jlem*^	Targeted; intragenic deletion	129S4/SvJae	[40]
	*Rho*^*tm2*.*1KpaI*^	Targeted; p.Glu150Lys	129S6/SvEvTac, B6J, C57BL/6NTac	[41]
	*Rho*^*Tvrm1*^	Chemically induced (ENU); p.Tyr102His	B6J	[42]
	*Rho*^*Tvrm4*^	Chemically induced (ENU); p.Ile307Asn	129S4/SvJae, B6J	[42]
	*Rho*^*Tvrm144*^	Chemically induced (ENU); p.Trp35Arg	B6J	[24],[25]
**Syndromic**				
ALMS, MIM# 203800	*Alms1*^*tvrm102*^	Chemically induced (ENU); c.1080+2T>C	B6J	this report
	*Alms1*^*foz*^	Spontaneous; intragenic deletion	NOD	[43]
	*Alms1*^*Gt(XH152)Byg*^	Gene trap: Insertion of gene trap vector	129P2/OlaHsd, B6J	[44]
	*Alms1*^*L2131X*^	Chemically induced (ENU); p.Leu2131*	C57BL/6^d^, NOD	[45]
	*Alms1*^*m1Btlr*^	Chemically induced (ENU); p.Gln1028*	B6J	
LKPAT, MIM# 615651	*Clcn2*^*nmf289*^	Chemically induced (ENU); p.Gly482Val	BALB/cByJ	this report
	*Clcn2*^*nmf240*^	Chemically induced (ENU); p.Gln355*	B6J	[46]
	*Clcn2*^*tm1Mlv*^	Targeted; intragenic deletion	C57BL/6 and unspecified	[47]
	*Clcn2*^*tm1Tjj*^	Targeted; intragenic deletion	129X1/SvJ, 129S1/Sv, C57BL/6^d^	[48]
MDDGA5, MIM# 613153	*Fkrp*^*tvrm53*^	Chemically induced (ENU); p.Ile356Thr	B6J	this report
	*Fkrp*^*tm1Itl*^	Targeted; p.Pro448Leu	C57BL/6NTac, 129S6/SvEvTac	[49]
	*Fkrp*^*tm1Qll*^	Targeted; p.Glu310Ter	C57BL/6NTac, 129S6/SvEvTac	[49]
	*Fkrp*^*tm1Scbr*^	Targeted; p.Tyr307Asn	C57BL/6^d^	[50]

Ocular disease classification, affected genes, mutations, and strain background of new TVRM models and previously reported non-transgenic strains that carry allelic mutations.

^a^ LCA, Leber congenital amaurosis; RP, retinitis pigmentosa: ALMS, Alström syndrome: LKPAT, leukoencephalopathy with ataxia; MDDGA5, muscular dystrophy-dystroglycanopathy (congenital with brain and eye anomalies), type A, 5.

^b^ Alleles designated *nmf* were initially identified in the Neuroscience Mutagenesis Facility (NMF) at JAX [51] and subsequently transferred to the TVRM program for completion.

^c^ Protein changes, p; cDNA changes, c; see http://varnomen.hgvs.org for a description of variant nomenclature.

^d^ The C57BL/6 substrain used in the referenced report was not indicated.

### Nonsyndromic ocular disease models

#### Aryl hydrocarbon receptor-interacting protein-like 1, *Aipl1*^*tvrm119*^ and *Aipl1*^*tvrm127*^

Mutations in the gene encoding aryl hydrocarbon receptor-interacting protein-like 1 (*AIPL1*) cause Leber congenital amaurosis 4 (LCA4), early-onset cone-rod dystrophy and juvenile retinitis pigmentosa [52], [53], which share the same disease phenotype designation at the Online Mendelian Inheritance in Man website (MIM 604393; www.omim.org). Mutations in *AIPL1* are responsible for 4–8% of total LCA cases [54]. The early onset and severity of *AIPL1* mutations suggest that developmental defects contribute to the disease. The human *AIPL1* gene is highly expressed in the retina and pineal gland, and the corresponding mouse *Aipl1* gene is highly expressed in the retina and RPE (biogps.org).

The AIPL1 protein consists of an N-terminal FK506 binding protein (FKBP) homology domain and a C-terminal domain containing three tetratricopeptide protein interaction motifs [52], suggesting a role in protein transport or chaperone activity [55]. AIPL1 co-chaperone activity is supported by its binding to heat shock proteins HSP70 and HSP90, and by co-suppression of protein aggregation in the presence of HSP70 [56]. In rod and cone cells, AIPL1 functions as a chaperone in phosphodiesterase PDE6 assembly, an activity that depends on binding of farnesylated PDE6α subunits to a unique polypeptide insert within the FKBP homology domain of AIPL1 [32], [57], [58]. The toxicity of AIPL1 variants has been proposed to result from an increase in cGMP due to the loss of PDE6 in rod photoreceptor cells [22]. However, toxicity may also be influenced by alteration of other AIPL1 activities, such as its function in cones to promote the stable expression of guanylate cyclase RetGC1 [57], or its interaction with the NUB1 regulator of the NEDD8 ubiquitin-like protein modification machinery, potentially regulating cell cycle progression during photoreceptor cell maturation [59].

Ramamurthy et al. [32] described the retinal phenotype of a targeted global knockout model, *Aipl1*^*tm1Visu*^, which develops early onset pan-retinal photoreceptor degeneration and concomitant reduction in ERG amplitude. Photoreceptor outer segments (OSs) were disorganized and the outer nuclear layer (ONL) exhibited a single layer of nuclei at postnatal day 18 (P18). A second global knockout of *Aipl1* (*Aipl1*^*tm1Mad*^) showed similarly rapid photoreceptor degeneration, initiating at P12 [30]. In contrast, a knockdown of *Aipl1* in *Aipl1*^*tm1Till*^ mice did not show a significant effect until three months of age [31].

**Fig 1:**
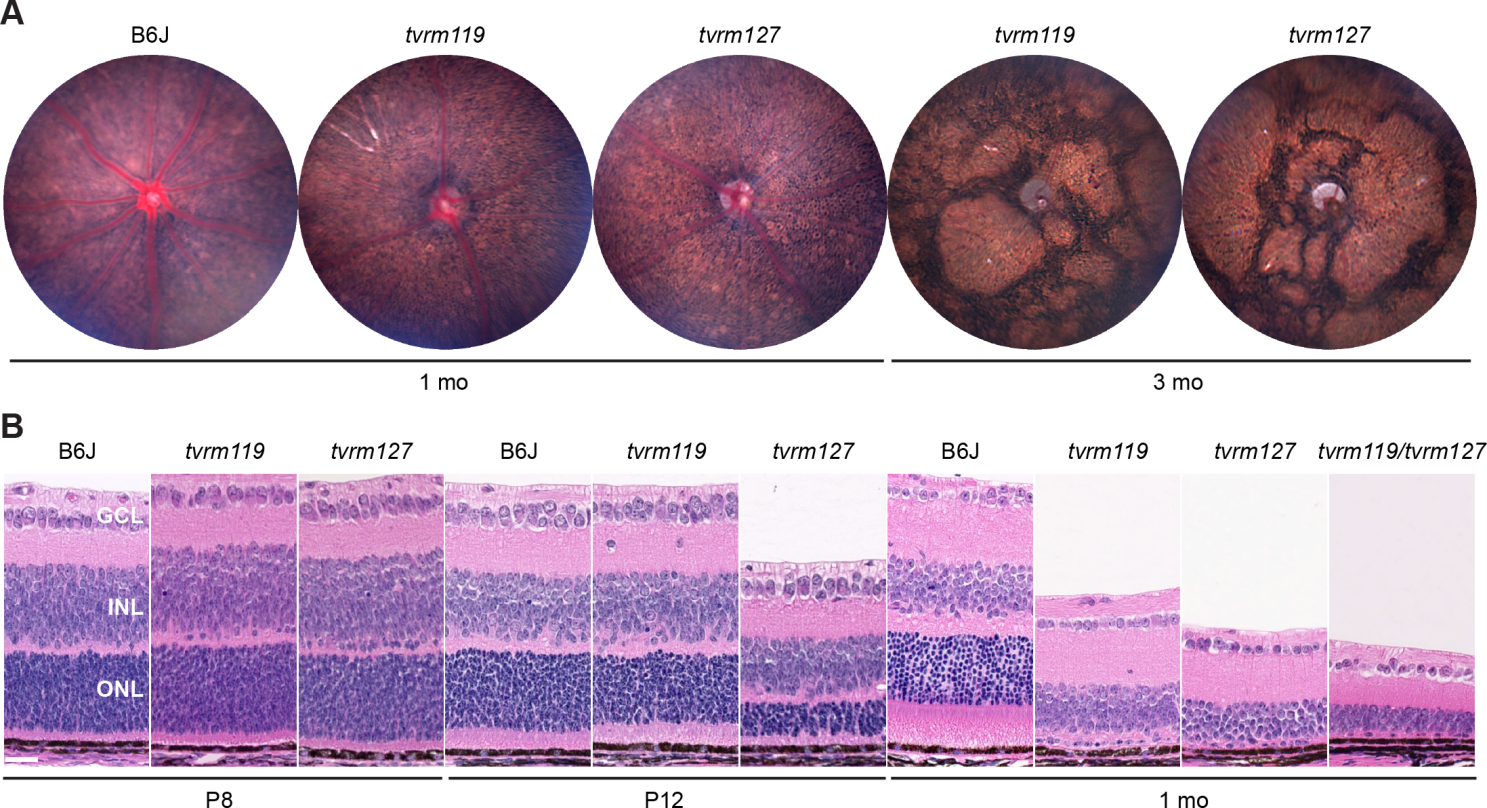
Rapid photoreceptor degeneration in *Aipl1* mutants. (A) Fundus photographs of B6J control and homozygous *Aipl1*^*tvrm119*^ (*tvrm119*) or *Aipl1*^*tvrm127*^ (*tvrm127*) mice at one and three months (mo) of age. (B) Retinal sections stained with hematoxylin and eosin (H&E) obtained from B6J control, *tvrm119*, and *tvrm127* mice at P8 (n = 3, 4 and 4, respectively), P12 (n = 3, 2 and 3, respectively) and one month (n = 3, all strains) of age visualized by light microscopy. An allele test of *Aipl1*^*tvrm119*^*/Aipl1*^*tvrm127*^ compound heterozygous (*tvrm119/tvrm127*) mice showed rapid loss of photoreceptors at one month of age (n = 8). GC, ganglion cell; INL, inner nuclear layer; ONL, outer nuclear layer. *Bar*, 25 μm.

We have identified two new *Aipl1* alleles, *Aipl1*^*tvrm119*^ and *Aipl1*^*tvrm127*^. Indirect ophthalmoscopy revealed that both lines had a fundus presentation consistent with early onset photoreceptor degeneration, characterized by a grainy fundus appearance with attenuated blood vessels that progressed to a patchy retina consistent with glial scarring. These observations were confirmed by fundus imaging. In mice with a grainy phenotype, fundus imaging clearly resolved individual retinal pigment epithelium (RPE) cells (Fig 1A), as reported among other retinal degeneration mutants [60], [61]. The causative mutation in both lines mapped to Chromosome (Chr) 11. *Aipl1* mutations were identified by direct sequencing of retinal cDNA and genomic DNA within the minimal region defined by mapping. In *Aipl1*^*tvrm119*^ mice, a nucleotide substitution in a splice donor site, c.276+5G>A, is predicted to lead to a loss of exon 2, resulting in an in-frame deletion of polypeptide residues 33–92 (p.Val33_Ile92del). This deletion encompasses most of the FKBP homology domain of AIPL1 (S1 Fig) disrupting the farnesyl binding motif. Interestingly, the pVal33_Ile92del transcript is a normal splice isoform in humans, present as 3.4% of AIPL transcripts [62]. In addition, two rare variants in the corresponding human splice donor site (rs150097891, c.276+1G>A and c.276+2T>C) also produce pVal33_Ile92del as the major transcript in *in vitro* splicing assays [62]. In *Aipl1*^*tvrm127*^ mice, a G to T transversion at nucleotide 266 (c.266G>T) is predicted to lead to a missense substitution, p.Cys89Phe (S1 Fig). An LCA-associated variant at the same position of human AIPL1, Cys89Arg, abolishes farnesyl-Cys binding without grossly affecting AIPL1 folding [58]. The *Aipl1*^*tvrm119*^ and *Aipl1*^*tvrm127*^ mutations are both predicted to reduce AIPL1 activity due to the loss of Cys89, which is expected to disrupt the binding of farnesylated PDE6α during assembly of the PDE6 complex.

Photoreceptor degeneration occurs rapidly in both *Aipl1*^*tvrm119*^ and *Aipl1*^*tvrm127*^ homozygotes, but *Aipl1*^*tvrm119*^ mutants have a noticeably less severe phenotype than *Aipl1*^*tvrm127*^ mutants at early time points (Fig 1B). At P8, the earliest time point sampled, the photoreceptor layers of both mutants appeared similar to B6J controls. By P12, *Aipl1*^*tvrm119*^ mutants displayed similar OS lengths compared with control mice and 9–10 layers of photoreceptor nuclei, whereas *Aipl1*^*tvrm127*^ mutants had markedly shorter OSs and only 7 layers of nuclei. By one month of age, both mutants and compound heterozygotes had only one layer of photoreceptor nuclei, which exhibited histological characteristics of cone nuclei. Compound heterozygous mice carrying both alleles also displayed rapid degeneration, confirming that the mutations are alleles of the same gene (Fig 1B). The more rapid degeneration in *Aipl1*^*tvrm127*^ mice is surprising given that the *Aipl1* missense mutation in these mice would be predicted to be less disruptive than the deletion in *Aipl1*^*tvrm119*^ mice. A possible explanation could be a potential residual production of correctly spliced transcript from the *Aipl1*^*tvrm119*^ allele.

**Fig 2:**
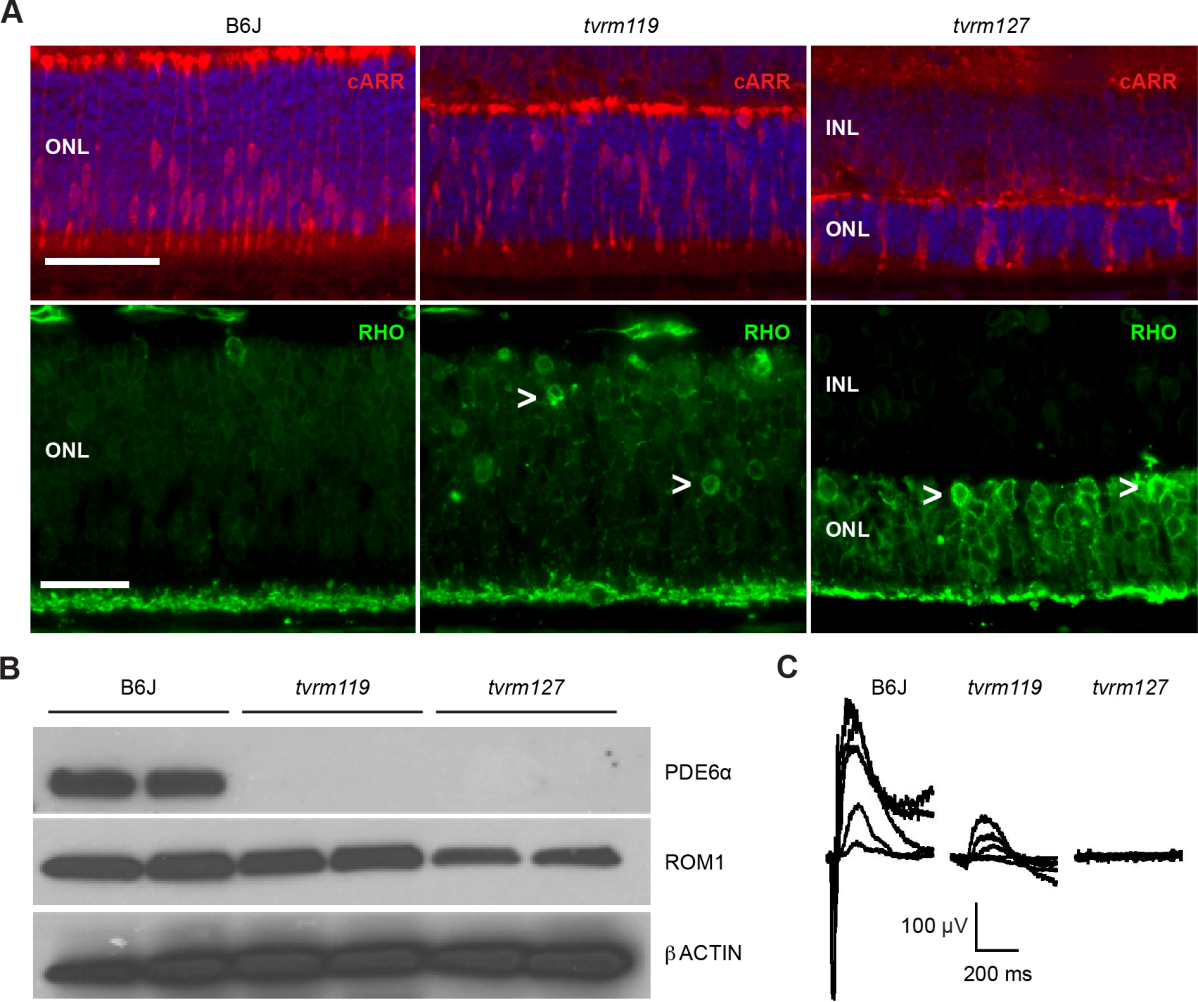
IHC, western analysis and ERG of homozygous *Aipl1* mutants. (A) Immunostaining of *Aipl1*^*tvrm119*^ (n = 2) and *Aipl1*^*tvrm127*^ (n = 3) and B6J control (n = 3) retinas at P12. Lower panel: mislocalization of rhodopsin (*green*) to the ONL was observed in both mutants. Upper panel: anti-cone arrestin (*red*) staining shows uniform cells bodies on the scleral side of the photoreceptor ONL while cell bodies were found scattered throughout the ONL in *Aipl1*^*tvrm119*^ mutants. Nuclear staining with DAPI (*blue*) shows a more pronounced photoreceptor degeneration in *Aipl1*^*tvrm127*^ mutants. *Bar*, 50 μm. (B) Western analysis at P10–12 with ROM1 antibody, an OS marker, shows similar relative expression in homozygous *Aipl1*^*tvrm119*^ (n = 2) and B6J control (n = 4) mice, but reduced expression in homozygous *Aipl1*^*tvrm127*^ mice (n = 4). Analysis with PDE6α antibody shows loss of PDE6α in *Aipl1*^*tvrm119*^ and *Aipl1*^*tvrm127*^ mutant mice. β-actin was probed as a loading control. (C) Dark- and light-adapted ERG analysis of P18 *Aipl1*^*tvrm119*^ (n = 2) and *Aipl1*^*tvrm127*^ (n = 6) mutants and B6J control (n = 2) mice.

Immunostaining with rhodopsin and cone arrestin antibodies in P12 retinal sections showed mislocalized rod pigments and displaced cones in homozygotes of both mutant strains (Fig 2A). By western analysis, similar levels of ROM1 were observed in *Aipl1*^*tvrm119*^ and control retinas (Fig 2B), consistent with the normal OS appearance in *Aipl1*^*tvrm119*^ mice at this age (Fig 1B). Analogous to histological findings, OS loss in *Aipl1*^*tvrm127*^ mice was reflected in a reduction in ROM1 levels. Furthermore, despite the presence of OSs in homozygous *Aipl1*^*tvrm119*^ mice at P12, a reduction in PDE6α level was observed in both *Aipl1*^*tvrm119*^ and *Aipl1*^*tvrm127*^ retinas. The loss of photoreceptor nuclei and outer segments were associated with a significant reduction in ERG response amplitudes at the earliest ages examined. At P18, the dark-adapted ERG responses of homozygous *Aipl1*^*tvrm119*^ mice were present but markedly reduced in amplitude, while those of *Aipl1*^*tvrm127*^ mice were reduced to the background noise level (Fig 2C).

These results indicate that the new alleles of *Aipl1* affect the same gene, cause a severe defect in PDE6 biogenesis, and result in retinal degeneration during early postnatal development. The differential effects of *Aipl1*^*tvrm119*^ and *Aipl1*^*tvrm127*^ on early degeneration may be useful for delineating the role of the AIPL1 FKRP domain in mammalian rod and cone photoreceptor cell development. One of the challenges in understanding genotype-phenotype relationships in *AIPL1-*associated retinal degeneration has been the high number of possibly benign polymorphisms in the human gene [63], which make it difficult to decide which variants should be targeted to create mouse models of the disease. The Cys89Phe mutation of *Aipl1*^*tvrm127*^ mice is informative in this regard, as homozygous mice bearing this mutation appear to mimic the effects of the Cys89Arg mutation in LCA patients. *Aipl1*^*tvrm119*^ mice can also serve as a novel model for the human splice variants c.276+1G>A and c.276+2T>C. Further study of these strains may improve understanding of how defects in AIPL1 binding of Cys-farnesylated proteins contribute to photoreceptor cell degeneration.

#### Retinitis pigmentosa GTPase regulator interacting protein 1, *Rpgrip1*^*tvrm111*^

Mutations in human *RPGRIP1* are associated with LCA6 (MIM 613826) [54], [64] and cone-rod dystrophy 13 (CORD13, MIM 608194) [65]. Both diseases are similar forms of early-onset photoreceptor degeneration, but cone-rod dystrophy is distinguished by early loss of cone cells throughout the retina, accompanied by retinal pigment deposits at the fovea, which is rich in cone cells. The RPGRIP1 protein localizes to the photoreceptor cell connecting cilium [34] and is required for normal OS disc initiation and morphogenesis [33], [34]. RPGRIP1 is important for the ciliary localization of proteins necessary for normal function of the connecting cilium, such as NPHP4 [66] and SPATA7 [67].

**Fig 3:**
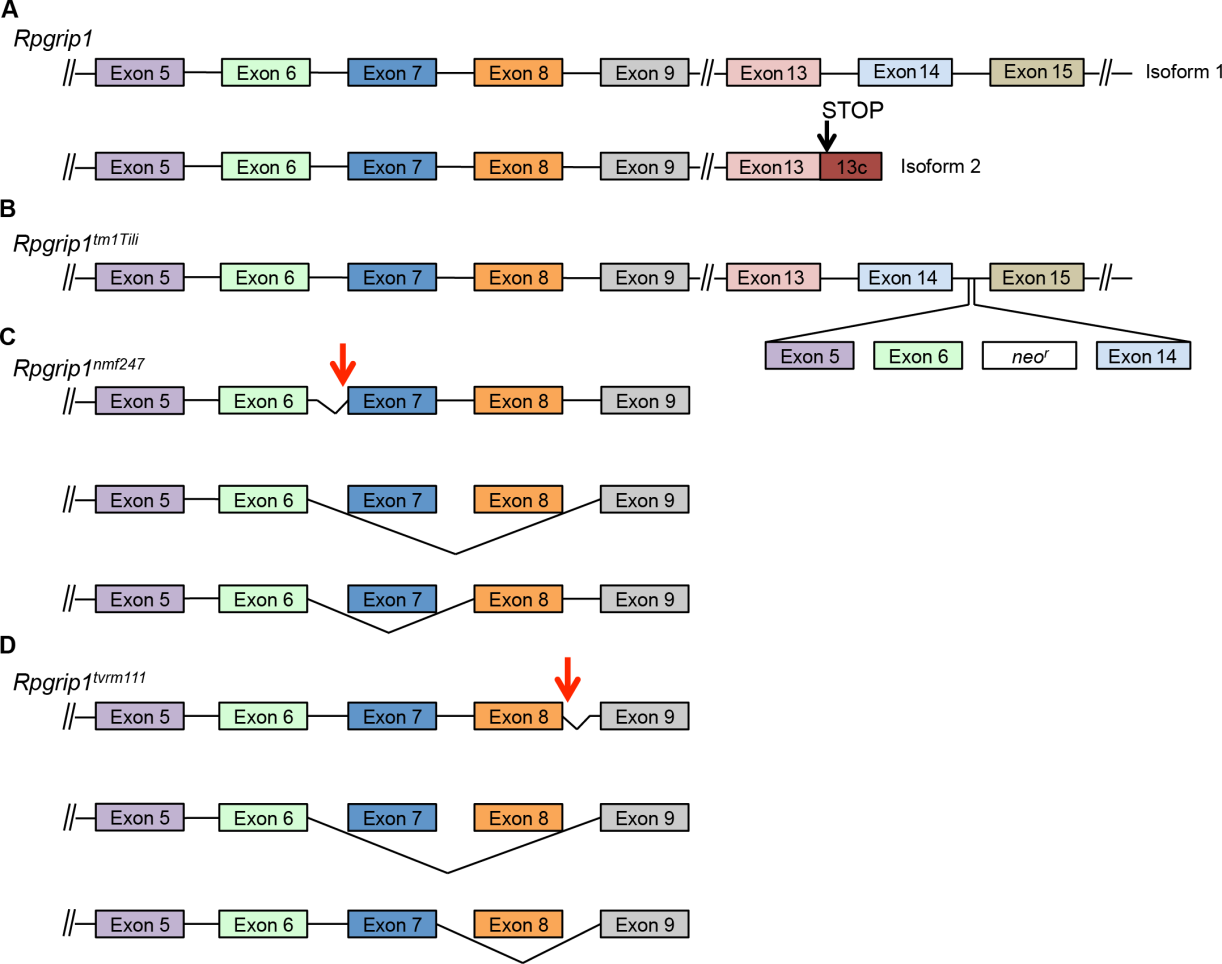
mRNA isoforms in targeted and chemically induced *Rpgrip1* murine models. (A) Wild-type (WT) mice produce a full-length *Rpgrip1* transcript, isoform 1 (NM_023879.3), and a short transcript, isoform 2 (NM_001168515.1) that contains an extension of exon 13 leading to an early termination. (B) The targeted insertion of a large cassette in *Rpgrip1*^*tm1Tili*^ mice between exons 14 and 15 leads to undetectable full-length *Rpgrip1* but does not affect the shorter *Rpgrip1* isoform. (C). *Rpgrip1*^*nmf247*^ splice-donor mutation produces multiple isoforms that lead to premature termination of both WT *Rpgrip1* isoforms. Aberrant splicing into intron 6 generates a 96 bp insertion, but is predicted to terminate at the mutation site because it generates a stop codon (*top*). Skipping of exon 7 (*middle*) or exons 7 and 8 (*bottom*) results in a frame-shift that is predicted to lead to a premature termination in exon 9. (D) *Rpgrip1*^*tvrm111*^ splice-donor mutation in intron 8 produces multiple transcripts that lead to premature termination or in-frame deletion of WT *Rpgrip1* isoforms. In one transcription product detected by cDNA sequencing, aberrant splicing to a cryptic site 214 bp downstream in intron 8 (*top*) is predicted to yield isoforms that encode an additional eight amino acid residues and result in premature termination. In a second product, skipping of exons 7 and 8 results in a frame-shift that is predicted to lead to a premature termination in exon 9 (*middle*). In a third product, skipping of exon 8 leads to an in-frame deletion of eight residues (*bottom*). This splicing event is predicted to occur in both full-length and short *Rpgrip1* isoforms. Exons are colored boxes while intron sequence is represented by lines. Exons and introns are not to scale. *Red arrows* denote location of mutations.

Two previously described mouse models bearing mutations in *Rpgrip1* (*Rpgripl*^*tm1Tili*^ and *Rpgrip1*^*nmf247*^) both lead to rapid photoreceptor degeneration [33], [34], but with differences in phenotype that have been attributed to differential effects on mRNA splicing [33]. At least two *Rpgrip1* splice variants have been validated (Fig 3A), a long transcript (isoform 1) and a short transcript (isoform 2) [68]. The site in which *Rpgrip1* is disrupted differs in the two mutant models. In *Rpgripl*^*tm1Tili*^ mice, the mutation targets intron 14, potentially allowing production of transcripts similar to isoform 2 [33] while abolishing isoform 1 (Fig 3B). A shortened RPGRIP1 polypeptide was detected in this strain [33]. By contrast, in *Rpgrip1*^*nmf247*^ mice, the mutation disrupts the splice acceptor site of intron 6 (Fig 3C), leading to undetectable levels of RPGRIP1 protein of any length [33]. The mutant alleles differentially affect OS formation, leading either to OS dysmorphogenesis in *Rpgripl*^*tm1Tili*^ mice [34] or OS absence in *Rpgrip1*^*nmf247*^ mice [33].

**Fig 4:**
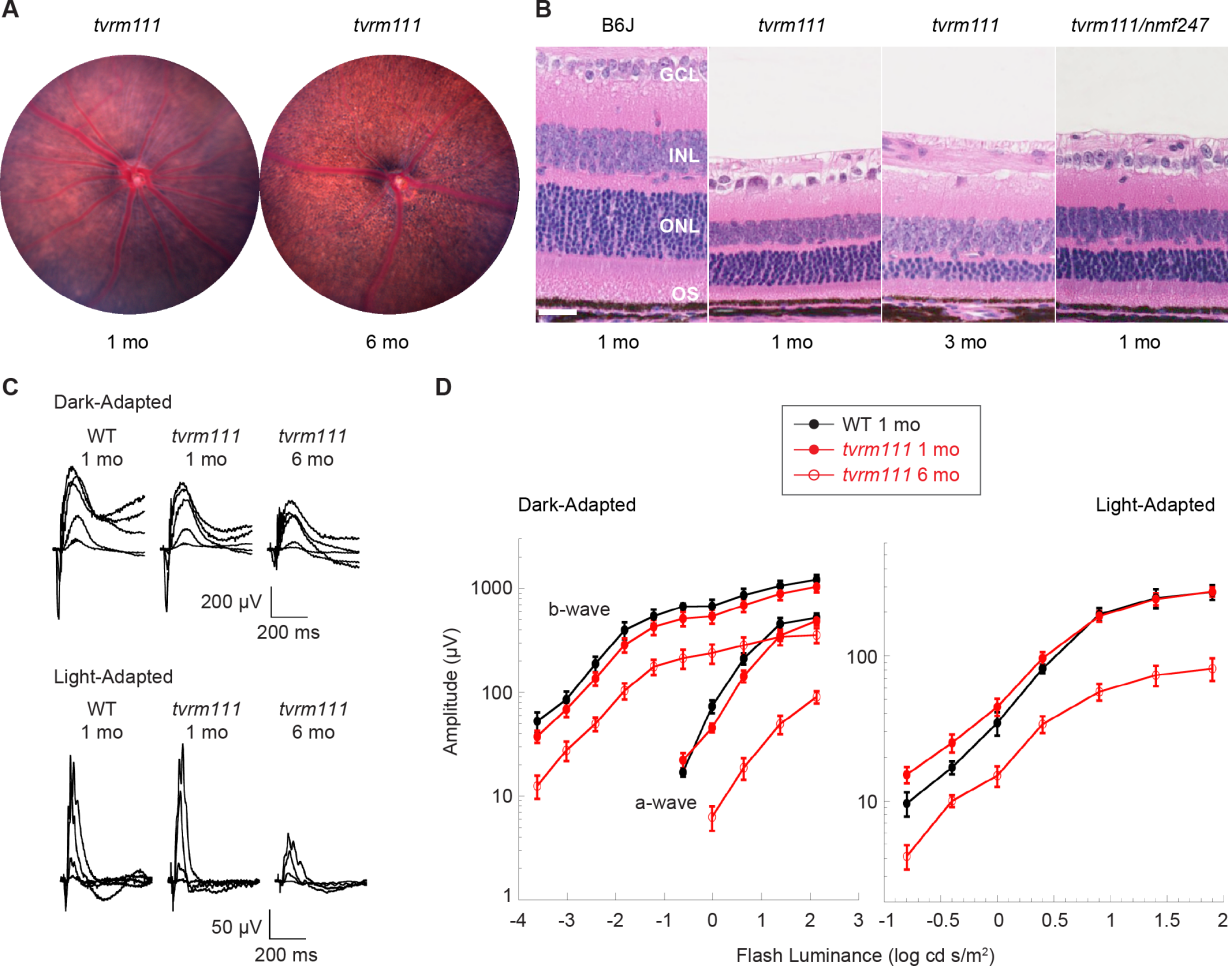
Progressive photoreceptor degeneration in *Rpgrip1* mutants. (A) Fundus images of homozygous *Rpgrip*^*tvrm111*^ mice (*tvrm111*) at 1 and 6 months of age. (B) Retinal sections stained with H&E obtained from B6J at 1 month of age (n = 3) and *Rpgrip1*^*tvrm111*^ at 1 (n = 3) and 3 (n = 6) months of age, or compound heterozygous *Rpgrip1*^*tvrm111*^*/Rpgrip1*^*nmf247*^ (*tvrm111/nmf247*) mice at 1 month (n = 3) of age, and visualized by light microscopy. Retinal layers are labeled as in Fig 1B. *Bar*, 25 μm. (C) Dark- and light-adapted ERG responses in *Rpgrip1*^*nmf247*^ homozygotes at 1 (n = 6) and 6 (n = 5) months of age and in B6J (n = 4) controls at 1 month of age. (D) Dark- and light-adapted ERG response amplitudes at varying illuminance determined from the samples tested as in C. Values indicate mean ± SEM.

**Fig 5:**
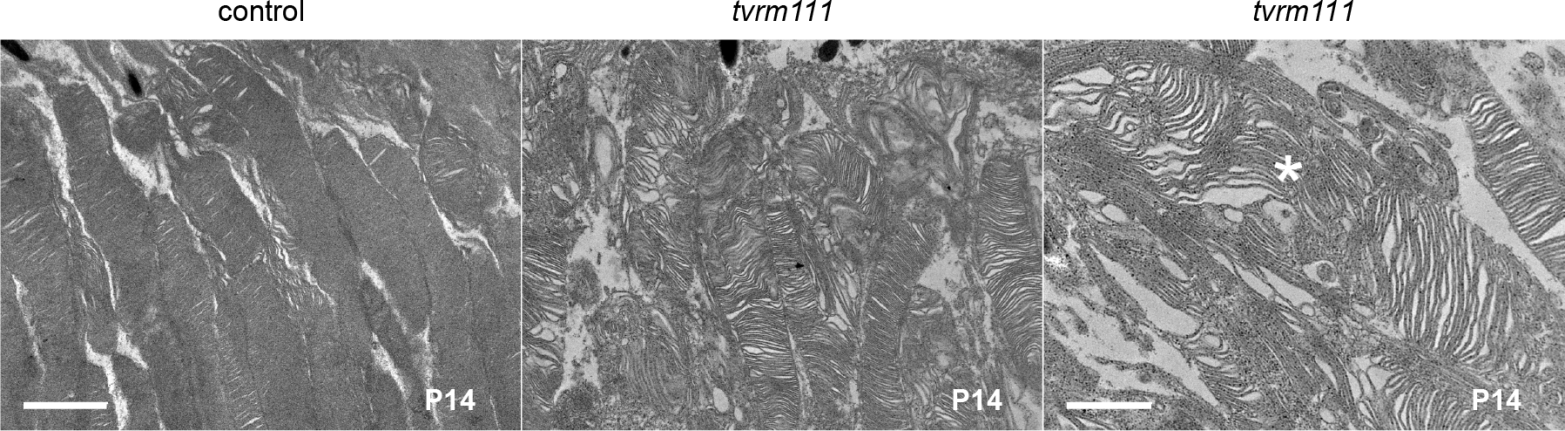
Ultrastructural analysis of OS alterations in *Rpgrip1*^*tvrm111*^ mice. Transmission electron micrographs at P14 show disorganization of photoreceptor OS discs in *tvrm111* (n = 3) compared to control (n = 3) mice. As can be seen at higher magnification, some discs have a vertical arrangement within the OS (*right*, *asterisk*) similar to the *Rpgrip*^*tm1Tili*^ mutant [34]. *Bar*, *left* panel, 2 μm; *right*, *1* μm.

Our new allele of *Rpgrip1* on the B6J background, *tvrm111*, is caused by a point mutation in the splice donor site, c.813+1G>A, in intron 8 of the gene (Fig 3D). This mouse was identified based on a grainy phenotype by indirect ophthalmoscopic examination. Fundus images at one month of age were similar to those of B6J mice (compare Fig 4A with Fig 1A), but at six months of age individual RPE cells were observable, as is characteristic of retinal degeneration (Fig 4A). In the *Rpgrip1*^*nmf247*^ mutant, the ONL was reduced to 3–4 nuclei at three weeks of age [33]. By contrast, the photoreceptor layer in homozygous *Rpgrip1*^*tvrm111*^ mice was reduced to 3–4 nuclei at three months of age (Fig 4B), similar to that of *Rpgrip*^*tm1Tili*^ mice [34] and indicating a slower rate of degeneration. Mice that are compound heterozygotes for *tvrm111* and *nmf247* alleles display photoreceptor degeneration, indicating that the alleles affect the same gene (Fig 4B). ERG responses of homozygous *Rpgrip1*^*tvrm111*^ were reduced progressively at one and six months of age corresponding to the slow rate of photoreceptor loss in the mutants relative to controls (Fig 4C). At both ages, mutant dark- and light-adapted ERGs were reduced to similar extents. Unlike *Rpgrip*^*nmf247*^ mice, which rarely form OSs, transmission electron microscopy (EM) analysis demonstrated that fully mature OSs were formed in *Rpgrip1*^*tvrm111*^ mice (Fig 5). However, the arrangement of the OS discs was frequently disorganized, often forming a vertical arrangement similar to that observed in the *Rpgrip*^*tm1Tili*^ strain [34]. This result indicates that the *Rpgrip1*^*tvrm111*^ mutation interferes with proper disc alignment during OS morphogenesis.

As the mutation in the splice donor site could affect splicing events and result in different splice variants, mRNA from *Rpgrip1*^*tvrm111*^ was used to generate cDNA for further examination. Three transcripts were identified (Fig 3D), including one in which exon 8 was deleted in-frame (Fig 3D; cDNA sequences are available through the data repository). Transcripts that include this splicing event are predicted to encode RPGRIP polypeptides carrying an internal deletion of eight amino acids that may retain partial or full function. By contrast, all transcripts from the *Rpgrip1*^*nmf247*^ allele indicated the presence of frameshifts predicted to cause early truncation of the encoded protein [33], which may account for the more severe phenotype observed in *Rpgrip1*^*nmf247*^ compared to *Rpgrip1*^*tvrm111*^ mice. This allelic series of *Rpgrip1* mutations underscores the importance of splice variants on disease presentation and may assist in furthering our understanding of the role of RPGRIP1 in photoreceptor cells.

#### Rhodopsin, *Rho*^*Tvrm334*^

The gene encoding the opsin moiety of rhodopsin (RHO) is the first for which mutations were linked to an inherited eye disease, retinitis pigmentosa (RP) [69], [70]. Since that initial observation, hundreds of different *RHO* mutations have been associated with dominant or recessive RP (MIM 713731) and another ocular disease, congenital stationary night blindness, autosomal dominant 1 (CSNBAD1; MIM 610445) [71], [72]. RHO, a major component of the rod photoreceptor cell, is a G protein-coupled receptor that assembles in OS disc membranes with other components of the visual transduction machinery to carry out the first steps in vision. The RHO polypeptide chain, opsin, includes seven transmembrane α-helices that surround a covalently-bound 11-*cis* retinal cofactor, which is required for light detection.

A number of transgenic mouse models for *RHO-*variant associated disorders have been generated to assess underlying pathologic mechanisms [73], but interpreting these models is obscured by variable *Rho* expression due to gene dosage or transgene location effects [74]. Missense *Rho* strains have been previously created by knock-in mutagenesis [37–39], [41] or by forward genetics using ENU-mutagenesis [24], [25], [35], [36], [42] (see also Table 1). Many of the missense mutations available in mice listed in Table 1 have already been characterized extensively *in vitro* in the context of the human or bovine opsin polypeptides [75–84]. Homozygous mutations that disrupt 11-*cis* retinal binding, protein folding, and trafficking through the secretory pathway lead to a rapid loss of photoreceptors within the first month after birth [35], [36], [39]. Other point mutations in the mouse *Rho* gene, notably the Trp35Arg, Tyr102His and Ile307Asn variants encoded by the *Rho*^*Tvrm144*^, *Rho*^*Tvrm4*^, and *Rho*^*Tvrm1*^ alleles, respectively (Table 1), are characterized by normal biosynthesis and transport of RHO to the OS under lighting conditions used in conventional mouse husbandry and display photoreceptor degeneration only upon exposure to bright light for two minutes or more [24], [25], [42]. These light-hypersensitive RHO variants may cause disease by a mechanism other than cytotoxicity arising from aberrant folding during transit through the secretory pathway. Thr4Arg and Thr17M RHO, which also cause light-hypersensitivity in animal models [85–87], have an abnormal photochemical reaction pathway *in vitro* [75], [82], [88] that may account for their light-induced toxicity *in vivo*. Additionally, rapid degeneration induced by a short exposure to bright light in *RHO* Thr4Arg dogs proceeds by a fundamentally different disease mechanism than by induction of the unfolded protein response [89].

**Fig 6:**
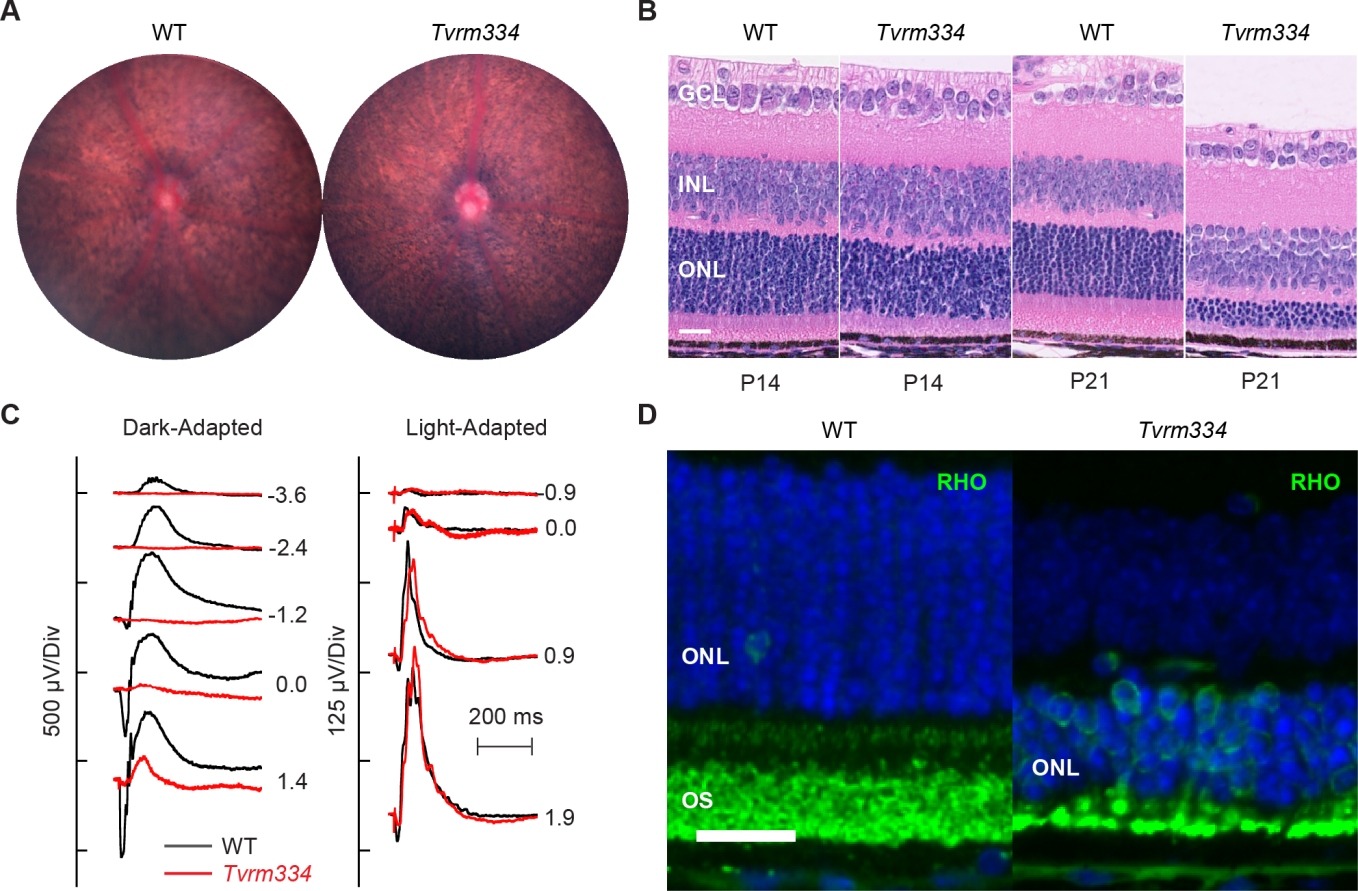
Clinical and histological alterations in heterozygous *Rho*^*Tvrm334*^ mice indicate a rapid, early onset degeneration. (A) Fundus photographs at P21 indicate atypical RPE characteristic of the grainy retinal appearance. (B) Retinal sections stained with H&E obtained from WT littermate control and heterozygous *Rho*^*Tvrm334*^ (*Tvrm334*) mice at P14 (n = 4, both genotypes) and P21 (n = 5 and 3, respectively) as visualized by light microscopy. Retinal layers are labeled as in Fig 1B. *Bar*, 25 μm. (C) ERGs recorded at P21 from WT littermate control (*black*; n = 4) and heterozygous *Rho*^*Tvrm334*^ (*red*; n = 4) mice. (D) Immunostaining of heterozygous *Tvrm334* mutants and WT littermate controls with anti-rhodopsin (green) at P21 (n = 3, both genotypes). RPE, retinal pigment epithelium. ONL, outer nuclear layer; OS, outer segment. *Bar*, 20 μm.

The *Tvrm334* mutant was identified by indirect ophthalmoscopy based on a grainy fundus and atypical RPE appearance in heterozygous matings, suggesting a dominant inheritance of retinal degeneration. Based on the dominant inheritance pattern and rapid degeneration, retinal cDNA of a *Tvrm334* mutant was sequenced and a *Rho* missense mutation, c.533A>G, was identified, corresponding to a p.Tyr178Cys substitution in rhodopsin. Phenotypic characterization was carried out using heterozygous *Rho*^*Tvrm334*^ mice. Fundus imaging revealed RPE cells consistent with retinal thinning (Fig 6A). These mice exhibit rapid photoreceptor degeneration, showing a slightly thinned outer nuclear layer at P14 and progressing to 3–4 rows of photoreceptor nuclei at P21 (Fig 6B). At P21, cone ERGs are comparable to those of control littermates (Fig 6C, *left*). In comparison, rod-mediated function is markedly reduced and the residual responses seen to strong stimulus flashes likely reflect dark-adapted cone ERGs (Fig 6C, *right*). Immunostaining with rhodopsin antibody revealed RHO mislocalization to the photoreceptor cell soma in heterozygous *Tvrm334* mutants at P14 (Fig 6D), indicating a trafficking defect. Homozygous *Tvrm334* mice exhibited a more rapid degeneration, also characterized by substantial opsin mislocalization (S3 Fig).

The RHO Tyr178Cys mutation in *Rho*^*Tvrm334*^ mice is identical to a variant identified in human RP [90–92]. Based on expression studies of bovine opsin variants in cultured cells, this mutation results in retention of the opsin polypeptide in the endoplasmic reticulum, accompanied by a decreased yield of the protein and no detectable binding of 11-*cis* retinal [75], [76]. Tyr178 is part of a two-stranded β-sheet in an extracytoplasmic (intradiscal) loop (S1 Fig), which includes an essential disulfide-bonded cysteine (Cys187) and has been proposed to constitute a lid or plug that acts to retain 11-*cis* retinal [93–95]. Thus, mutation of Tyr178 in mice may cause disease by virtue of a defect in 11-*cis* binding and/or protein trafficking.

The *Rho*^*Tvrm334*^ allele is the fourth dominant *Rho* mutation identified through the TVRM program (Table 1). Light-inducible mutants *Rho*^*Tvrm1*^, *Rho*^*Tvrm4*^, and *Rho*^*Tvrm144*^ may be useful for identifying the rapid molecular changes in photoreceptor OSs that occur upon bright illumination. Examination of three mutants in the allelic series (*Rho*^*Tvrm1*^, *Rho*^*Tvrm144*^, and *Rho*^*Tvrm334*^) on the same B6J genetic background as two other reported ENU-induced *Rho* mutations, (*Rho*^*Noerg1*^ and *Rho*^*R3*^) may help to identify shared and independent pathways of photoreceptor damage that are thought to underlie autosomal dominant RP caused by *RHO* variants [82], [89], [96–98]. These dominant *Rho* mutations may also be suitable for pre-clinical tests of CRISPR-based gene therapy of somatic tissue in the context of autosomal dominant RP, as successfully demonstrated for dominant muscular dystrophy in mice [99–101].

### Syndromic ocular disease models

#### Alström syndrome 1, *Alms1*^*tvrm102*^

*ALMS1* mutations are responsible for human Alström syndrome (ALMS, MIM 203800), a multisystemic and progressive disorder characterized by early ocular phenotypes of nystagmus and photophobia and progressive cone-rod photoreceptor degeneration [102]. Additional clinical features include hearing loss, truncal obesity, type 2 diabetes, cardiomyopathy, steatohepatitis, kidney disease and genitourinary defects [103]. The ALMS1 protein is a component of centrosomes of dividing cells and the ciliary basal body. Given the central role of the connecting cilium in the formation and maintenance of rod and cone OSs, mutations in *ALMS1* are expected to exhibit defects in photoreceptor biogenesis and function.

Mouse models for Alström syndrome have been characterized to varying extents and include *Alms1*^*foz*^ [43], *Alms1*^*Gt(XH152)Byg*^ [44], *Alms*^*L2131X*^ [45], and *Alms1*^*m1Btlr*^ (mutagenetix.utsouthwestern.edu) mice. Obesity, a cardinal feature of Alström syndrome, is observed in all of these models. Cochlear degeneration and metabolic anomalies, such as hyperinsulinemia and renal and liver dysfunction, were documented in *Alms1*^*foz*^ and *Alms1*^*Gt(XH152)Byg*^ mice, and retinal degeneration was reported in *Alms*^*L2131X*^ and *Alms1*^*Gt(XH152)Byg*^ mice.

**Fig 7:**
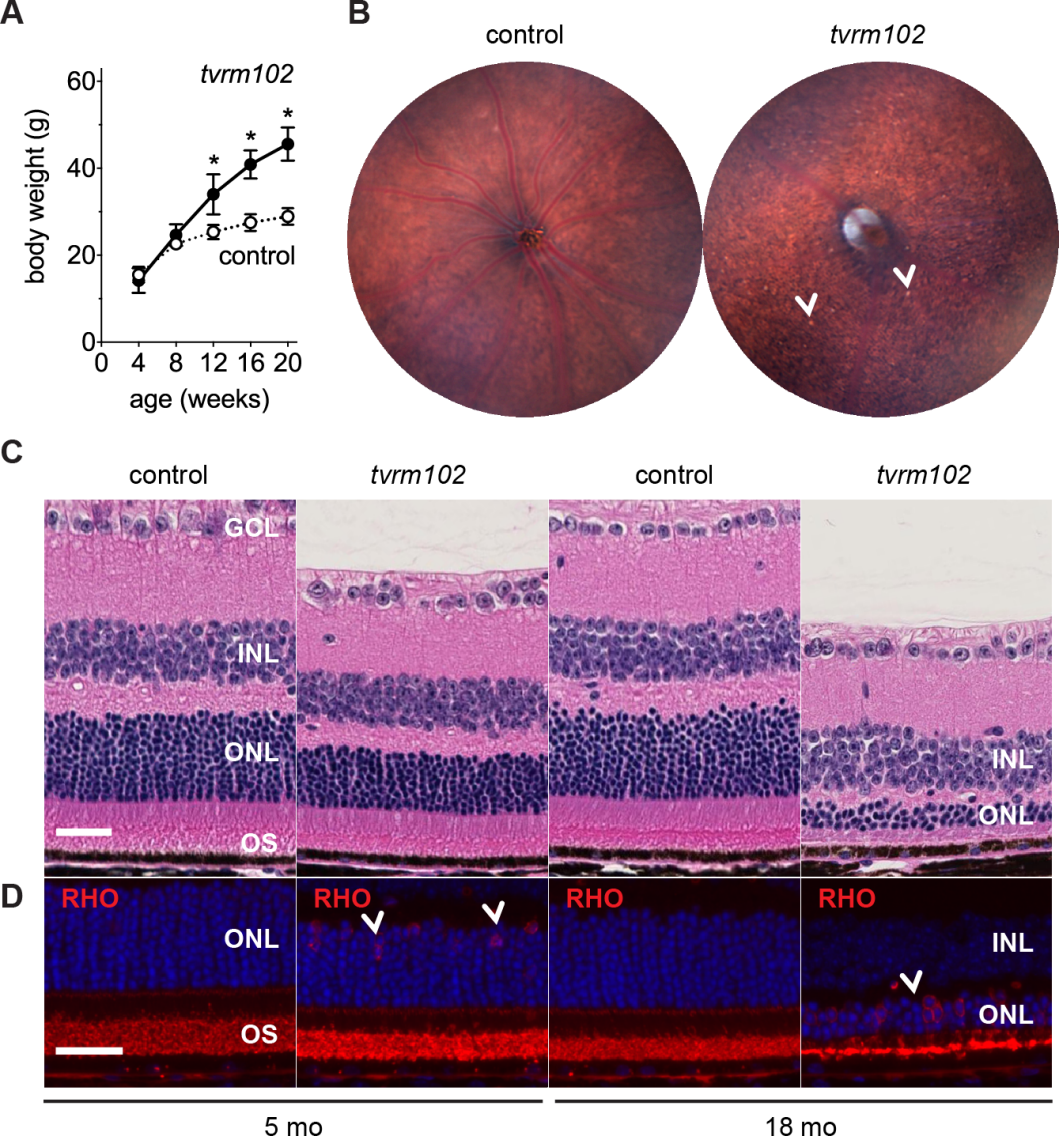
*Alms1*^*tvrm102*^ recapitulates clinical phenotypes observed in patients with Alström syndrome. (A) Obesity is an early clinical feature of homozygous *Alms1*^*tvrm102*^ (*tvrm102*) mice (n = 6). Control mice (n = 10) include WT and heterozygous littermates. Graph shows that male mutant mice begin weight gain between 8–12 weeks of age. Values indicate mean ± standard deviation; *, *p*<0.0001. (B) Fundus images of WT littermate control and homozygous *Alms1*^*tvrm102*^ mice at 15 months of age. *Arrowheads*, small bright spots. (C) Brightfield microscopy of H&E stained retinal sections showing a slow progression of photoreceptor loss in *Alms1*^*tvrm102*^ mutants at 5 (n = 4) and 18 months (n = 3) of age, and WT and heterozygous controls at 5–6 months (n = 3) and 18 months (n = 3) of age. Retinal layers are labeled as in Fig 1B. (D) Immunostaining with rhodopsin (*red*) in homozygous *Alms1*^*tvrm102*^ mice at 5–6 (n = 3) and 18–20 (n = 3) months of age. Arrowheads show mislocalized rhodopsin in photoreceptor somata in the ONL. Controls at the same ages are as in C (n = 3). *Bars*, 30 μm.

The *Alms1*^*tvrm102*^ mutant was discovered in an ENU line co-segregating for obesity and pan-retinal spots (determined by body weight measurements and fundus examination, respectively). A recessive mode of inheritance was determined and a genome wide scan of *tvrm102* (B6J × DBA/2J) F_2_ intercross progeny revealed linkage to Chr 6 in a critical interval overlapping the *Alms1* locus. Allele complementation testing with *Alms1^Gt(XH152)Byg^* indicated that *tvrm102* was a new allele of *Alms1*, as all compound heterozygous mice became obese and exhibited photoreceptor degeneration. Sequencing *Alms1* in *tvrm102* mutant cDNA revealed a T>C point mutation in the exon 6 splice donor site unmasking a cryptic splice site 120 bases downstream (S3 Fig). Abnormal weight gain in *Alms1*^*tvrm102*^ mice begins between 8 and 12 weeks of age (Fig 7A). Compared to littermate controls (Fig 7B, *left*), fundus images of homozygous *Alms1*^*tvrm102*^ mice showed a grainy and atypical RPE appearance at 5 months of age that eventually included small bright spots by 15 months of age (Fig 7B, *right*). *Alms1*^*tvrm102*^ mice show a marginal thinning of the retinal ONL at five months of age, with a further slow progressive degeneration with ~25% of photoreceptors remaining at 18 months of age (Fig 7C). A trafficking defect of rhodopsin is shown by opsin mislocalization to the photoreceptor cell soma in *tvrm102* mutants at 5 and 18 months of age (Fig 7D).

These studies indicate that homozygous *Alms1*^*tvrm102*^ mice exhibit an obesity and retinal degenerative phenotype characteristic of Alström syndrome. The rate of photoreceptor degeneration in these mice is similar to that of *Alms1^Gt(XH152)Byg^* mice [44]. However, *Alms1*^*tvrm102*^ mice may represent a better model of the human disease, as they lack exogenous gene trap DNA sequences within the *Alms1* locus.

#### Chloride channel 2, *Clcn2*^*nmf289*^

Human *CLCN2* variants lead to leukoencephalopathy with ataxia (LKPAT; MIM 615651) [104], [105]. A subset of individuals with *CLCN2-*associated LKPAT also have visual acuity defects or chorioretinopathy, suggesting a role of the CLCN2 protein in tissues of the posterior eye. CLCN2 (also known as ClC-2) is a member of a family of ion channels and exchangers that mediates the transport of chloride and possibly other anions, such as bicarbonate, in the context of plasma membrane excitability, ion and water homeostasis, cell volume regulation, and the function of lysosomes, endosomes and other intracellular organelles [106], [107].

Three *Clcn2* null alleles have been characterized in mice [46–48], including the targeted gene disruptions *Clcn2*^*tm1Tjj*^ and *Clcn2*^*tm1Mlv*^ and an allele obtained by chemical mutagenesis, *Clcn2*^*nmf240*^, which is predicted to cause early truncation of the encoded protein. Homozygous mice containing these alleles capture important aspects of the human disease, including progressive leukoencephalopathy and loss of photoreceptors [46], [47], [108], [109]. Photoreceptor degeneration is most rapid in the *Clcn2*^*nmf240*^ model, with only 1–2 layers remaining at P21 [46], in contrast to the *Clcn2*^*tm1Tjj*^ model that does not reach this stage until P31 [108]. As both are essentially null alleles, the altered degeneration rate may be caused by genetic background differences (B6J in [46] versus a mixed genetic background in [108]). It has been suggested that photoreceptor cell loss may be due to RPE defects, as *in situ* hybridization revealed *Clcn2* transcript in the RPE, INL and ganglion cell layer, but less prominently in the ONL [108]. In support of this idea, changes have been observed in the RPE of *Clcn2* mutant mice, including a decreased transepithelial voltage in explant measurements [108], a decreased ERG light peak amplitude in heterozygous *Clcn2*^*nmf240*^ mice [46], and increased apical microvilli length in homozygous *Clcn2*^*nmf240*^ mice [46]. These findings hint at an RPE-specific role of the CLCN2 protein that underlies the disease process, possibly its function in plasma membrane chloride transport [108] or in intracellular vesicular trafficking [110] which may influence the maintenance of microvilli [111].

**Fig 8:**
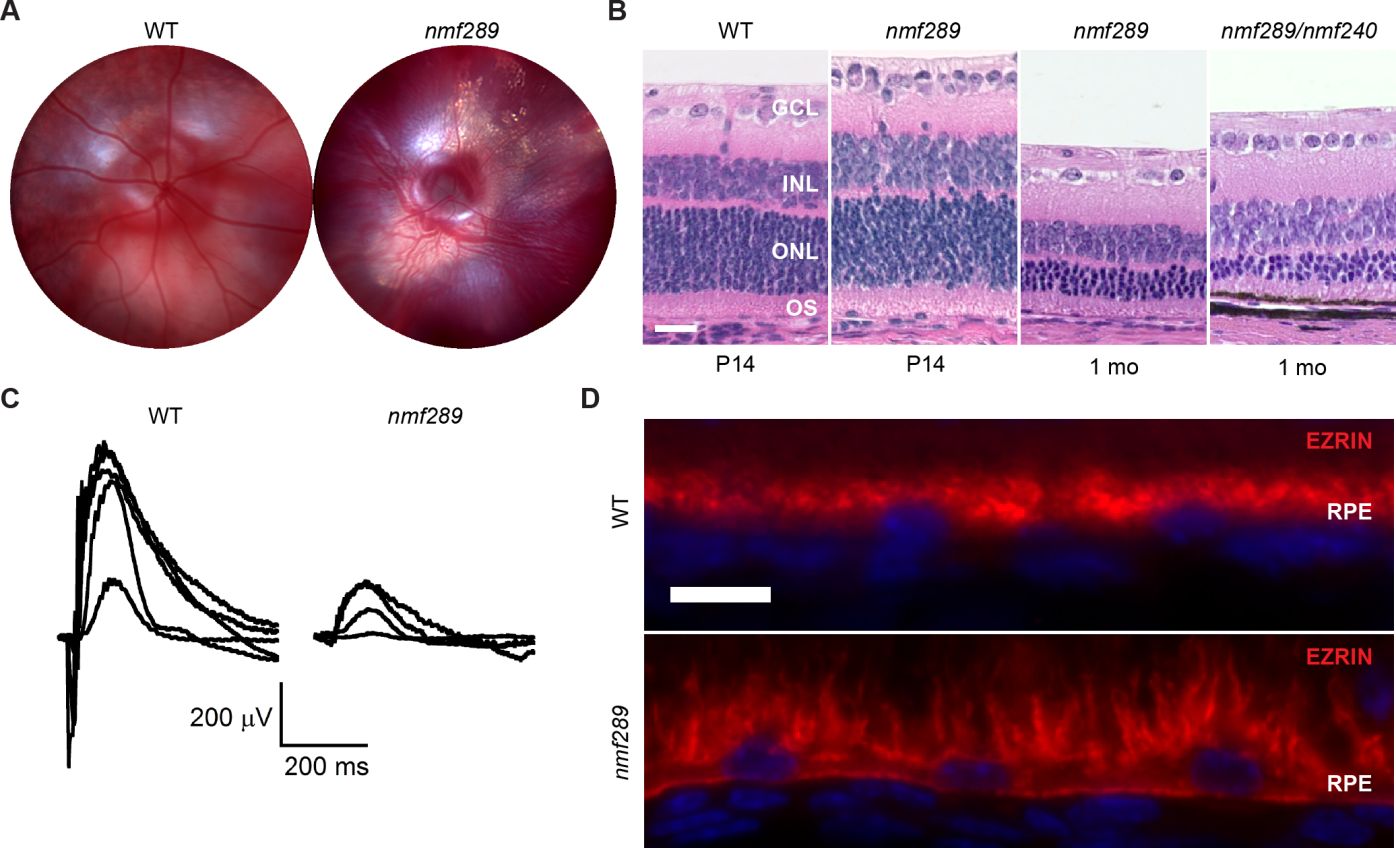
Gradual photoreceptor loss in homozygous *Clcn2*^*nmf289*^ mutants. (A) Fundus imaging of homozygous *Clcn2*^*nmf289*^ (*nmf289*) mice at 3 months of age indicates vascular atrophy compared to WT littermate controls. (B) Brightfield images of H&E-stained retinal sections of WT or heterozygous littermate controls at P14 (n = 5) and 1 month (n = 3) of age, and homozygous *Clcn2*^*nmf289*^ at P14 (n = 3) and at 1 month (n = 3) of age. Compound heterozygous *Clcn2*^*nmf289*^*/Clcn2*^*nmf240*^ (*nmf289/nmf240*) mice at P14 (n = 6) show similar ONL loss. Retinal layers are labeled as in Fig 1B. *Bar*, 20 μm. (C) Dark-adapted ERG series obtained from WT littermate control (*left*; n = 3) and homozygous and *Clcn2*^*nmf289*^ (*right*; n = 5) mice at 1 month of age (*left* to *right*). D. Immunostaining with anti-ezrin antibody shows elongated apical microvilli in homozygous *Clcn2*^*nmf289*^ mice (*bottom*; n = 3) compared to WT littermates (*top*; n = 3) at P14. *Bar*, 10 μm.

Affected *nmf289* mice, congenic on the BALB/cByJ albino background from the TVRM program, were identified based on fundus abnormalities that included vascular thinning, and a clearer definition of choroidal vessels, such as the long posterior ciliary arteries (Fig 8A). The trait mapped near the *Clcn2* locus and direct sequencing of retinal cDNA identified a c.1445G>T mutation. The *Clcn2*^*nmf289*^ mutation is predicted to lead to a missense mutation, p.Gly482Val. Based on protein modeling (S1 Fig), the mutation occurs in a transmembrane segment of the protein (helix 9, residues 465–488) that includes part of the chloride ion selectivity filter (residues 465–469). Substitution of Gly482, one of three conserved glycines on the face of helix 9 that contacts an adjacent intramembrane helix, might be expected to disrupt helical packing and thereby alter chloride conductivity.

In comparison to the null mutants, the *Clcn2*^*nmf289*^ mutation appears to have relatively mild effects on CLCN2 function, as 4–5 photoreceptor layers are retained at 1 month of age (Fig 8B). The *nmf289* allele affects the same gene as the previously described *nmf240* allele, as a similar extent of photoreceptor layer loss is observed in homozygous *Clcn2*^*nmf289*^ mice compared to *Clcn2*^*nmf289*^*/Clcn2*^*nmf240*^ compound heterozygous mice (Fig 8B, *last panel*). Dark-adapted ERG responses are substantially reduced in homozygous *Clcn2*^*nmf289*^ mice at 1 month of age, corresponding to the loss of rod photoreceptor cells (Fig 7C). As in *Clcn2*^*nmf240*^ mice [46], apical microvilli of RPE cells in homozygous *Clcn2*^*nmf289*^ mice appear elongated compared to those of control mice (Fig 8D).

**Fig 9:**
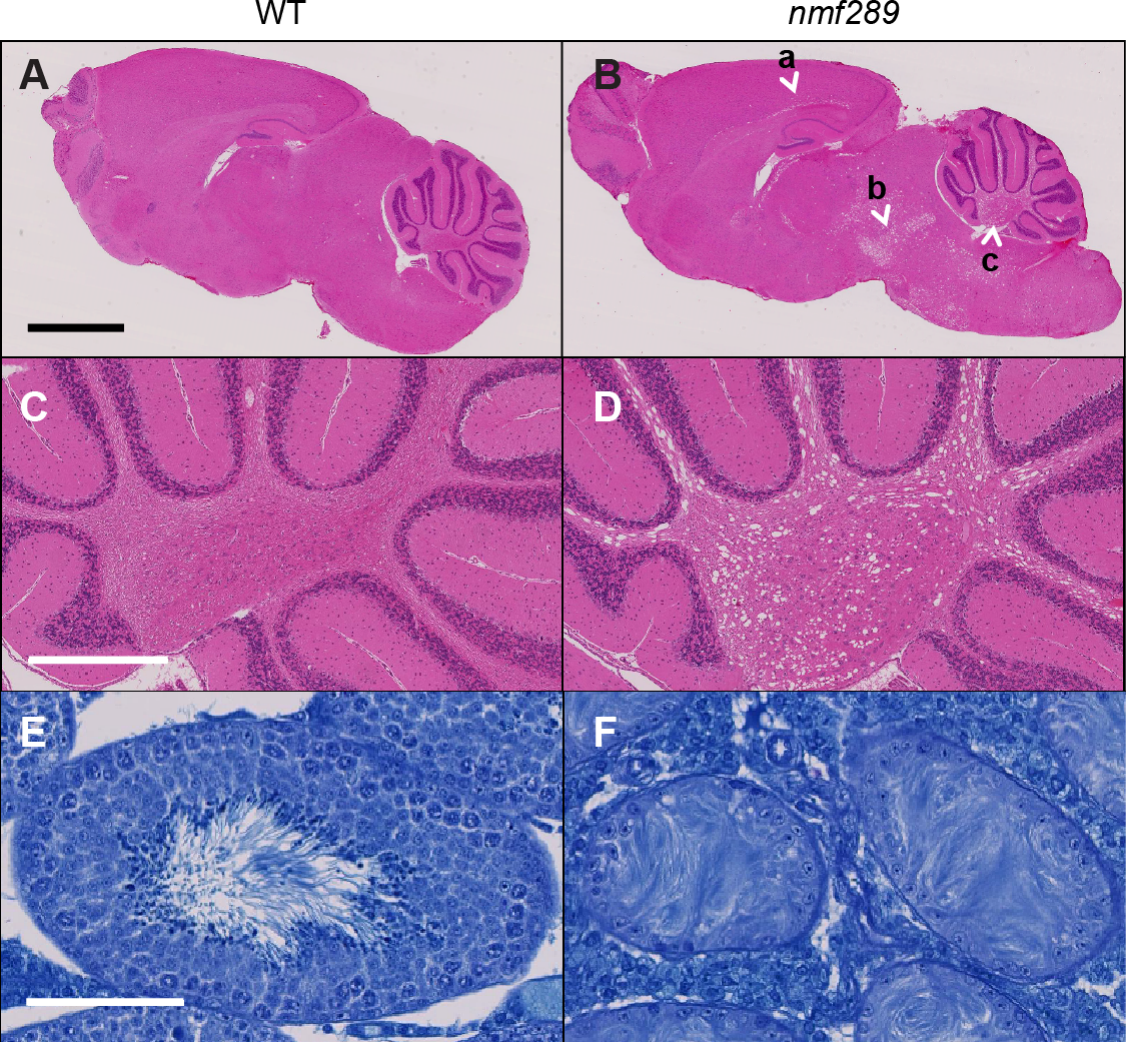
*Clcn2*^*nmf289*^ mice develop leukoencephalopathy and azoospermia. (A) Sagittal section of a control BALB/cByJ brain at 3–6 months of age stained with H&E (n = 4). (B) Similarly stained section of homozygous *Clcn2*^*nmf289*^ brain (mutant littermate; n = 5). Arrows depict marked vacuolization in the white matter tracts of the corpus callosum (a), brainstem (b) and cerebellum (c) of mutant mice. (C) Higher power image of the cerebellum of BALB/cByJ mice. (D) The cerebellum of homozygous *Clcn2*^*nmf289*^ mice exhibits vacuolization. (E) Toluidine blue-stained sections show normal spermatogenesis in BALB/cByJ testes (n = 4). (F) Atrophic seminiferous tubules were observed in testes of mutant littermates at 3–6 months of age (n = 4). *Bars*, (A-B) 2 mm, (C-D) 500 μm, (E-F) 100 μm.

Subclinical leukodystrophy, a progressive degeneration of white matter of the brain, and male infertility have been observed in *CLCN2* patients [105], [112] and in homozygous *Clcn2* mutant mice [46], [109]. To determine if *Clcn2*^*nmf289*^ mutants have leukoencephalic-like lesions, we examined H&E-stained sagittal sections of brains from BALB/cByJ controls and homozygous *Clcn2*^*nmf289*^ mice at 12 weeks of age (Fig 9A–9D). Areas of prominent vacuolization were observed along the white matter tracts of the corpus callosum, brain stem and cerebellum of *Clcn2*^*nmf289*^ mutant brains (Fig 9B and 9D). Male infertility was noted by the failure of homozygous *Clcn2*^*nmf289*^ males to sire offspring. Histological analysis revealed normal spermatogenesis in BALB/cByJ controls (Fig 9E), while *Clcn2*^*nmf289*^ mutants were azoospermic, lacking evidence of spermatocytes, spermatids or mature spermatozoa (Fig 9F).

The *Clcn2*^*nmf289*^ mutation results in the same phenotypes observed in other *Clcn2* mutant mice and in patients affected by *CLCN2* variants, thereby reinforcing the association of LKPAT disease phenotypes, including ocular defects, with mutations in this gene. The slower rate of photoreceptor degeneration in *Clcn2*^*nmf289*^ mice may provide a greater opportunity to explore underlying pathogenic mechanisms and to test therapeutic approaches. In future work, it may be of interest to characterize the electrophysiological consequences of the CLCN2 missense variant, as well as to examine possible alterations in the endosomal and exosomal pathways in RPE cells that may account for the increased length of apical microvilli, which may be relevant to the observed photoreceptor loss. Although the *Clcn2*^*nmf289*^ allele is available currently as a congenic on the BALB/cByJ strain genetic background, introgression onto B6 is possible and may be necessary to allow comparison of allelic differences.

#### Fukutin related protein 1, *Fkrp* ^*tvrm53*^

In humans, *FKRP* variants cause several types of congenital muscular dystrophy that differ in clinical presentation [113]: a severe form with brain and eye anomalies (type A5; MDDGA5, MIM 613153), a less severe form with or without mental retardation (type B5; MDDGB5, MIM 613153), and a limb-girdle form (type C5; MDDGC5, MIM 613153). *FKRP* is thought to influence the biosynthetic processing and/or activity of the dystrophin-associated glycoprotein complex (DGC), which connects the cytoskeleton to the extracellular matrix in muscle and at neuronal synapses [114], [115]. Specifically, *FKRP* is one of at least 17 genes implicated in the post-translational *O*-linked glycosylation of the transmembrane protein dystroglycan [116], [117], an αβ heterodimer that forms the core of the DGC. The FKRP protein localizes mainly to the Golgi apparatus [117], [118] but also associates with the DGC at the plasma membrane in muscle [115]. The protein is predicted to include short N-terminal transmembrane helix and Golgi-retention motif as well as a C-terminal nucleotidyltransferase domain (LicD) found in bacterial phosphorylcholine transferases and eukaryotic type-A glycosyltransferases involved in oligosaccharide modification (S1 Fig). Similar to LicD orthologs that use CDP-ribitol in the synthesis of bacterial capsule and cell wall glycopolymers, FKRP uses CDP-ribitol as a substrate for the transfer of ribitol phosphate onto *O*-mannosyl glycans of α-dystroglycan [119–121].

In mice, global knockout of *Fkrp* is embryonic lethal, leading to death by E12.5 [122], similar to the early lethality associated with homozygous *FKRP* null mutation [123]. A homozygous knock-in of the Tyr307Asn *Fkrp* allele is phenotypically similar to WT mice up to 6 months of age [124]. In contrast, mice homozygous with the same Y307N mutation and a neomycin cassette in intron 2 of the *Fkrp* gene die soon after birth [124]. The observed phenotype was consistent with the most severe MDDG in humans; mutant mice showed decreased muscle mass, and aberrancies of the inner limiting membrane (ILM) of the eye and of neuronal migration. Because *Fkrp* mRNA levels were WT-like in the former but 40% reduced in the latter, the investigators suggested that a hypomorphic *Fkrp* combined with a missense allele was necessary to give rise to the disease phenotype [124]. Using a similar strategy but a different allele, Chan et al. [122] reported widespread effects of the P448L allele in mice. Ocular abnormalities reported included optic nerve hypoplasia, abnormal optic disc morphology, variable eye size, abnormal ILM and retinal ganglion cell layer morphology, and thinning of the inner nuclear layer (INL) and ONL [122].

**Fig 10:**
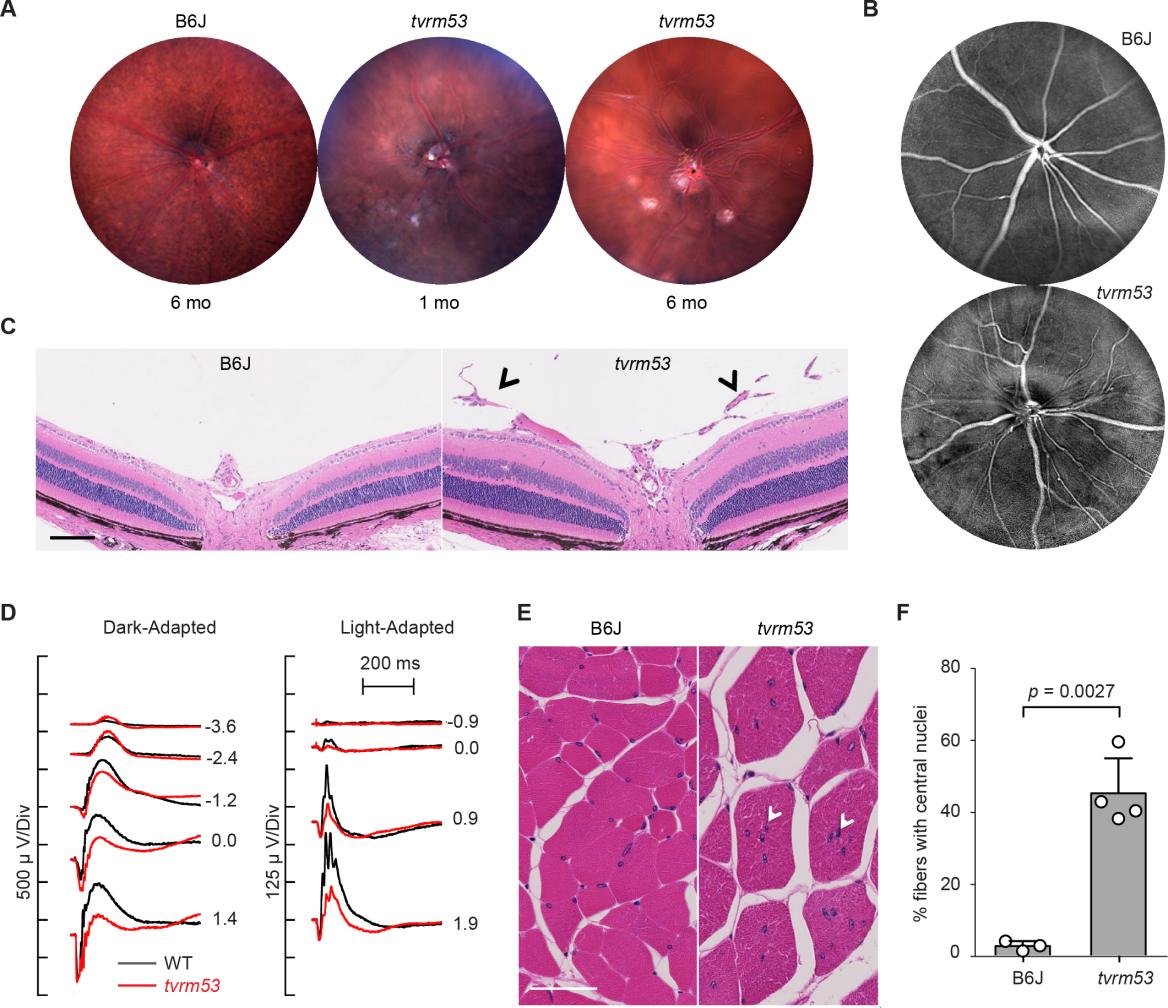
Aberrant clinical, histological and functional effects of the homozygous *Fkrp*^*tvrm53*^ allele. (A) Fundus of B6J control mice at 6 months and *Fkrp*^*tvrm53*^ (*tvrm53*) mutants at 1 and 6 months of age. (B) Fundus images of B6J control (*top*; n = 4) and *Fkrp*^*tvrm53*^ (*bottom*; n = 3) mice at 1 month of age, digitally processed to highlight vascular dysmorphology in the mutant strain. (C) Brightfield image of H&E stained-B6J control (n = 3) and *Fkrp*^*tvrm53*^ (n = 3) retina at one month of age. *Arrowheads* depict vascular abnormalities in the vitreous. *Bar*, 100 μm. (D) Dark-adapted (*left*) and light-adapted (*right*) ERGs obtained from a representative B6J control (n = 2) and *Fkrp*^*tvrm53*^ (n = 3) mouse at 7 weeks of age. (E) Spinal muscle showing normal distribution of nuclei at the periphery of muscle fibers in B6J mice (*left*; n = 3) and mislocalized nuclei in *Fkrp*^*tvrm53*^ mice (*right*; n = 4) at 12 months of age. *Bar*, 50 μm. (F) Quantitation of central nuclei in B6J (n = 3) and *Fkrp*^*tvrm53*^ mice (n = 4). Mean value and standard deviation is indicated.

The new allele of *Fkrp1*, *tvrm53*, was discovered based on fundoscopic observation of vitreal fibroplasia and retinal lesions (Fig 10A). The causative mutation encodes a missense substitution of isoleucine 356 for threonine within the putative nucleotidyltransferase domain of FKRP (S1 Fig). Homozygous *Fkrp1*^*tvrm53*^ mice exhibit similar ocular phenotypes as reported for FKRP-neo-P448L and *Fkrp*^*tm1Igl*^ mice. Digital processing of color fundus images indicated the presence of aberrant vascular formations (Fig 10B) similar to those observed in homozygous *Large*^*vls*^ and *Lama1*^*tvrm223*^ mutants, which also affect the DGC [125], [126]. Compared to B6J control animals (Fig 10C, *left*), vascular structures were found in the vitreous of homozygous *Fkrp1*^*tvrm53*^ mutants in regions where the ILM is disrupted (*arrowheads*, Fig 10C, *right*). Thinning of the peripheral INL and ONL was detected as early as 1 month of age, the earliest time point examined (not shown). RT-PCR analysis revealed a 1.53-fold higher level of *Fkrp* mRNA in *Fkrp1*^*tvrm53*^ compared to WT mice (relative normalized expression; 95% confidence interval, 1.20–1.95; *p* = 0.031; n = 4 and 5 for mutant and WT samples, respectively). This result indicates that the *Fkrp1*^*tvrm53*^ mutation does not abolish expression of the gene.

Ocular functional deficits were also apparent. Under dark-adapted conditions (Fig 10D, *left*), the amplitude of the ERG a-waves of homozygous *Fkrp1*^*tvrm53*^ mice is comparable to that of WT at high flash levels and somewhat larger than WT at intermediate flash levels. This increase reflects a slower than normal onset of the b-wave in *Fkrp1*^*tvrm53*^ mice. While the b-wave is somewhat larger than WT at low flash levels, b-wave amplitudes are decreased at the high flash levels. Cone ERGs are reduced in *Fkrp1*^*tvrm53*^ homozygotes across all stimulus conditions examined (Fig 10D, *right*). These ERG abnormalities are similar to, but less severe than, those reported in mouse models that disrupt proteins affecting assembly of the dystrophin-associated glycoprotein complex at the neuronal synapse, such as LARGE [125] or pikachurin [127].

To test the syndromic nature of the *Fkrp* mutation, muscles were examined for the presence of nuclei displaced from the periphery of individual muscle fibers, which are symptomatic of an attempt by degenerating muscle to regenerate lost fibers. Nuclei in spinal muscles of control B6J mice exhibited a normal distribution at the periphery of individual fibers (Fig 10E, *left*), but were frequently displaced from the fiber periphery in homozygous *Fkrp*^*tvrm53*^ mice (Fig 10E, *right*). Quantitation of these results indicated a statistically significant increase of ~17-fold in the percentage of fibers with central nuclei in *Fkrp*^*tvrm53*^ compared to B6J mice (Fig 10F). Centrally localized nuclei were observed in the anterior tibialis muscle of *Fkrp*^*tvrm53*^ mice (S4 Fig). Thus, severe muscular dystrophy is observed in homozygous *Fkrp*^*tvrm53*^ mice.

**Fig 11:**
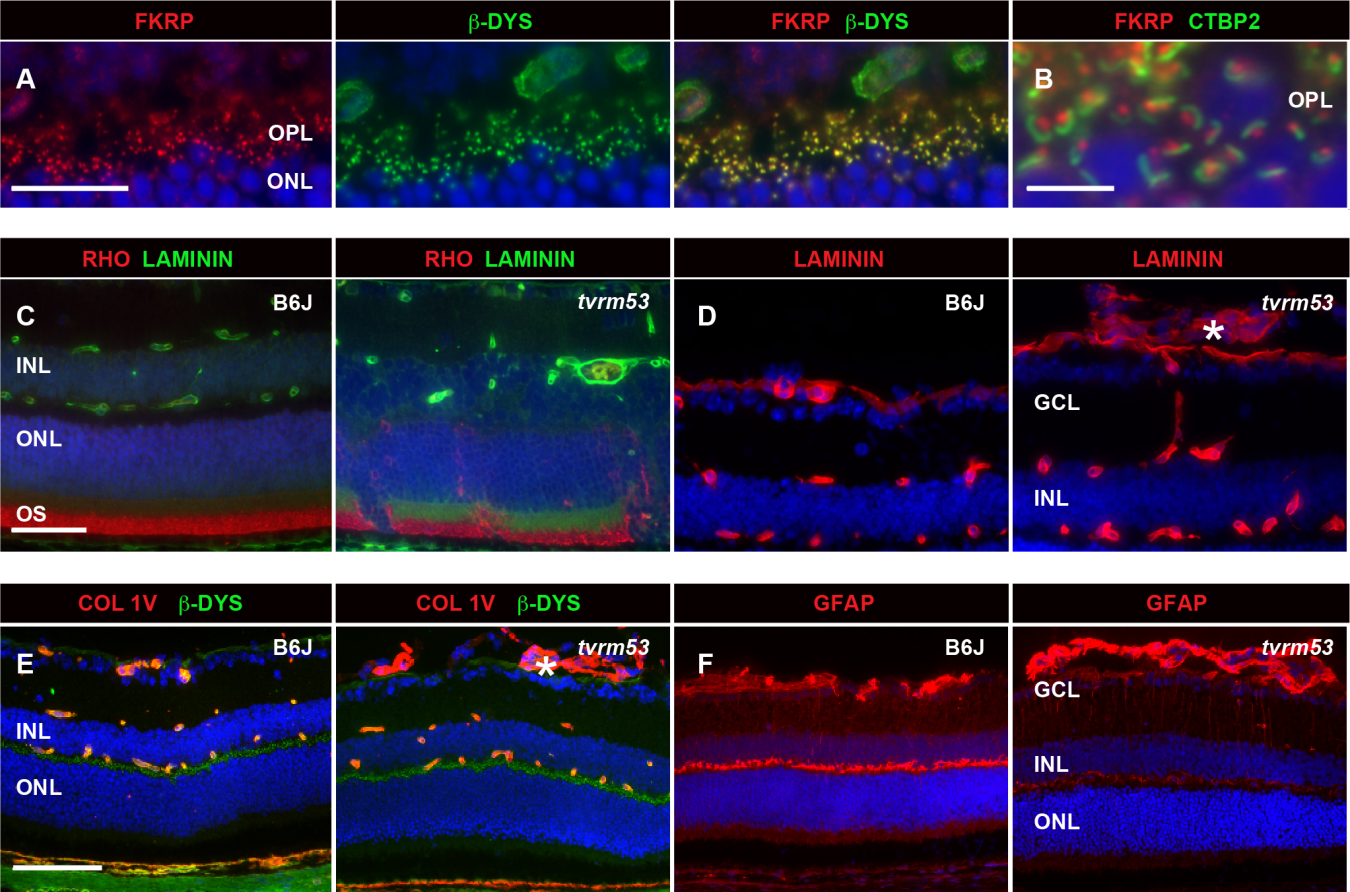
Marker analysis of homozygous *Fkrp*^*tvrm53*^ mice at 1–2 months of age. (A) Localization of FKRP in B6J control retina (n = 3). FKRP (*red*) colocalizes with β-dystrophin to synaptic processes in the OPL. (B) Co-staining of FKRP (*red*) with CTBP2 (*green*) shows FKRP surrounds the synaptic ribbons. (C) IHC of rhodopsin (*red*) and pan-laminin (*green*) in B6J control (n = 6) and homozygous *Fkrp*^*tvrm53*^ mice (n = 7). (D) IHC of pan-laminin in ocular cryosections of samples as in C. (E) IHC of collagen IV (COL IV, *red*) and β-dystroglycan (β-DYS, *green*) in cryosections (n = 5 and 6 for B6J and mutant mice, respectively). (F) Glial fibrillary acidic protein (GFAP) staining of retinal cryosections indicated Müller cell activation in homozygous *Fkrp*^*tvrm53*^ mice (n = 4) compared to B6J controls (n = 3). Retinal layers are labeled as in Fig 1B. *Asterisks*, abnormal vessels in (D, E). *Bars*, (A) 20 μm, (B) 5 μm, (C-D) 50 μm. (E-F) 100 μm.

Staining of retinas with anti-FKRP revealed co-localization withβ-dystroglycan in the synaptic processes of the OPL (Fig 11A), in close proximity to CTBP2, a major component of the synaptic ribbons (Fig 11B). Marker analysis showed mislocalization of rhodopsin in areas where protruding vessels disrupt the retinal lamination (Fig 11C). Both laminin (Fig 11D) and collagen IV (Fig 11E) are highly expressed in the abnormal vessels within the vitreous (*asterisks*). In addition, Müller cells were activated in *Fkrp*^*tvrm53*^ mutants as early as one month of age (Fig 11F).

Overall, our findings suggest that *Fkrp1*^*tvrm53*^ mutant mice model a severe form of congenital muscular dystrophy that includes ocular abnormalities (MDDGA5). In addition to known ocular defects in *Fkrp* mutant mice, delayed and decreased ERG responses were observed, suggesting a defect in coupling of photoreceptors to secondary neurons, and punctate FKRP staining was colocalized with β-dystroglycan at synapses within the OPL. These results indicate a requirement for FKRP at the photoreceptor synapse, a known site of accumulation of the dystroglycan-glycoprotein complex within the retina [128], [129]. The importance of mouse models that do not depend on a neomycin cassette to generate a hypomorphic allele, which may confound the phenotype by mechanisms unrelated to those present in human disease [130], has been emphasized [131]. Additionally, the availability of an allelic series on a constant genetic background may be critical for future studies, as the existence of possible genetic modifier is implied by differences in the phenotype of the same *Fkrp* allele when present on different genetic backgrounds [131]. Finally, characterization of α-dystroglycan oligosaccharide modifications in the retina and other tissues of *Fkrp*^*tvrm53*^ mutant mice may provide useful insights into aspects of FKRP enzymatic function in the native settings that are inaccessible *in vitro*.

## Conclusions

Classical (forward) genetics through mutagenesis and phenotypic screening is notable in its yield of novel “outside-the-box” genetic discoveries, in contrast to reverse genetics, an inherently biased strategy, in which mutations are targeted to specific genes and are most often null alleles. Chemical mutagenesis combined with forward genetic approaches has been widely used by the invertebrate research community for decades [132–136], and now successfully applied in mice as well. ENU-based screening programs have been used to generate new mouse models for a broad range of questions and applications, including metabolic bone disease [14], cancer [15], circadian biology [16], development [17], the nervous system [18], [19], fertility [20], the process of X-inactivation [21] and the immune system [22], [23].

The TVRM program [24], [25] applies this fruitful approach to the mouse visual system. Over the past decade, many new mouse mutants with ocular phenotypes have been identified in the TVRM program and subsequently developed with detailed characterization for distribution to the research community; 34 TVRM mutants are listed in S1 Table. Significantly, most TVRM mice are on the B6J background, or have been backcrossed to it, so the effects of segregating strain background genes or of the *Crb1*^*rd8*^ mutation found in B6N-derived models [7] on the expression of the disease phenotypes are less of a concern. Although ENU strains at early generations following mutagenesis contain multiple incidental mutations, most unlinked mutations are removed by multiple backcrosses to WT mice during the process of establishing heritability.

As many genes involved in eye development, function and maintenance have been documented, many of the TVRM mutants are new alleles of well-known genes. However, as the genotypes of these new ENU models are typically point mutations that are not predicted to be null alleles, the TVRM lines extend allelic series that may have the potential to expand our understanding of the functional domains of the affected proteins and of the related human conditions. For example, a recent model of congenital stationary night blindness, *tvrm27*, which carried a *Trpm1* point mutation, affected the pore-forming loop but did not alter the trafficking of the mutant protein leading to a phenotype distinct from that encountered in homozygous *Trpm1* knockout mice [137]. This fine-grained separation of phenotypes is not possible in knockout models, but is critical to allow features of the retinal phenotype related to protein absence to be separated from those related to abnormal protein function. Also, mutations may be within particular splice variants that have different functions, as was the case with *Rpgrip1*^*nmf247*^ versus *Rpgrip1*^*tm1Tili*^ mice, in which a short splice variant was found to be important for the initiation of OS formation while the longer splice variant was important for maintaining proper OS morphology [33].

A further advantage of the chemical mutagenesis approach is that exogenous DNA sequences are absent from the mutant strains produced. By contrast, traditional gene targeting approaches contain exogenous DNA sequences that usually include a selectable marker, which may or may not be removed from the targeted gene depending on the approach. For example, all of the targeted alleles and the gene trap allele listed in Table 1 include a neomycin cassette, except for four *Rho* alleles created by a targeting approach that removed the selection cassette (*Rho*^*tm1*.1(RHO*)Akgr^, *Rho*^*tm1*.*1Eye*^, *Rho*^*tm1*.*1KpaI*^ and *Rho*^*tm2*.*1KpaI*^). For *Aipl1*, *Rpgrip*, *Clcn2*, and *Fkrp*, the mutant alleles presented here are the only ones currently available that lack the neomycin cassette. The neomycin cassette in some mutant strains is thought to cause unanticipated effects, such as altering the expression of neighboring genes [138] or interfering with expression of a WT allele in heterozygotes [139]. Retention of the cassette may therefore complicate the interpretation of the mutant phenotype; indeed, complications were noted in the case of targeted *Fkrp* alleles as described above. Thus, alternate mutant strains, such as those generated by the TVRM program, which do not bear a selection cassette or any other exogenous sequences arising from targeted strain construction, may constitute more faithful models of human disease.

A key purpose of this report is to make the research community aware of new TVRM mutant mice on the B6J genetic background (with a single exception) that exhibit disease characteristics of inherited human ocular conditions. We recognize that concerted efforts by other laboratories will be required to evaluate these models completely and to advance our knowledge of the role of key molecules important in vision, ocular pathogenesis, and syndromic diseases. These mice are therefore made available to the research community.

## Materials and methods

### Ethics statement

All animal studies were performed according to protocols that were fully approved by the Institutional Animal Care and Use Committees of JAX (protocol number 99089, most recently renewed on November 21, 2014) and the Cleveland Clinic (protocol number 2014–1296, most recently approved September 25, 2014), and were in accordance with the “Guide for the Care and Use of Experimental Animals” established by the National Institutes of Health (1996, Revised 2011) and the Association for Research in Vision and Ophthalmology Statement for the Use of Animals in Ophthalmic and Vision Research. Veterinary care was provided under the supervision of the Comparative Medicine and Quality department at JAX and the Biological Resources Unit at the Cleveland Clinic.

### Mouse mutagenesis and husbandry

To generate TVRM mice, we administered N-ethyl-N-nitrosourea (ENU) in three weekly injections (80 mg/kg) to male B6J G_0_ mice. G_3_ offspring were generated using a three-generation mating scheme [24]. G_3_ mice were screened at 12 weeks, or at 24 weeks of age in order to enhance our ability to identify later onset diseases. The NMF mice were transferred to the TVRM program when the Neuromutagenesis Facility program ended. The generation and screening of mice were carried out in identical fashion as the TVRM program, with the exception that in one cohort, F_1_ BALB/CByJ and B6J mice were used as the G_0_ strain.

To determine if an abnormal ocular phenotype was heritable, mutant mice were outcrossed to WT B6J mice to generate F_1_ progeny, followed by intercrossing of the resultant F_1_ mice to generate F_2_ progeny. Screening protocols described below were used to examine both F_1_ and F_2_ mice. If F_1_ mice were affected, the heritability indicated a dominant trait. If F_1_ mice were not affected but ~25% of F_2_ mice were, the heritability indicated a recessive trait. Once the observed ocular phenotype was determined to be genetically heritable, mutants were backcrossed to the appropriate WT parental strain to remove unlinked ENU-induced mutations, in most cases, strain B6J, and maintained in the JAX Research Animal Facility. Mice were provided with NIH 6% fat chow diet and acidified water, with 12:12 hour dark:light cycle in pressurized individual ventilation caging which were monitored regularly to maintain a pathogen-free environment.

### Indirect ophthalmoscopy and fundus imaging

All mice were screened by indirect ophthalmoscopy using a procedure that has been previously described [140]. In brief, after dark adaption for a minimum of one hour, 1% atropine was used to dilate the pupil prior to examination by indirect ophthalmoscopy with a 60 or 78 diopter aspheric lens. The fundus was scored for abnormalities such as vascular attenuation, spots, depigmentation, lesions, etc. Representative images to capture the abnormal phenotypes as observed by fundus examination were obtained by imaging with a Micron III or IV fundus camera (Phoenix Research Laboratories, Pleasanton, CA). Fundus videos were acquired using the manufacturer’s software, registered, averaged, and processed using Fiji [141] as described [28], [142] to yield a single image for each eye examined. In Fiji, the Polynomial Shading Corrector was used with the following settings: Degree x, 2; Degree y, 2; Regularization percent of peak, 80. Brightness and contrast were adjusted manually in Fiji. To highlight vascular structures, the red channel of registered and averaged color fundus images was divided by the green channel using Fiji Image Calculator (32-bit output), the result was processed with Enhanced Local Contrast (default values, fast processing unselected), and brightness and contrast were adjusted manually.

### Electroretinography

A subset of mutagenized mice were also screened at JAX using an electroretinogram (ERG) protocol that has been previously described [26]. In brief, after at least two hours of dark adaptation mice were anesthetized with ketamine (16 mg/kg) and xylazine (80 mg/kg) diluted in normal saline. Strobe stimuli were presented to the dark-adapted eye and following at least 10 minutes of light adaption, cone ERG tests were carried out. ERG studies were also conducted at the Cleveland Clinic, using a protocol that has been described in detail [143].

### Genetic mapping

Genomic DNA was isolated from tail tips using a PCR buffer with nonionic detergents (PBND), which was adapted from a protocol described previously [144]. Tail tips were digested at 55°C in PBND containing Proteinase K overnight. Samples were heated to 95°C for 10 minutes and 1 μl of the DNA preparation was used in a 12 μl PCR reaction. Amplicons were visualized with ethidium bromide after electrophoretic separation on a 4% agarose gel.

For mapping purposes, phenotypically affected mice, homozygous for recessive mutations, were mated with DBA/2J or C3BLia mice. The resulting F_1_ offspring were intercrossed to generate F_2_ offspring. The resulting progeny were phenotyped by indirect ophthalmoscopy or ERG. DNA isolated from tail tips from at least 10 affected and 10 unaffected littermates was pooled and subjected to a genome-wide scan using 48–80 simple sequence length polymorphic markers distributed throughout the genome. Samples used in the DNA pools were tested individually to confirm map locations [145].

### Preparation of RNA samples and subsequent analysis

Total RNA was isolated from whole eyes and brains of affected mutants and B6J mice using TRIzol Reagent (Life Technologies) according to the manufacturer’s protocol. Total RNA was treated with RNase-free DNaseI (Ambion) and the quantity was determined using a NanoDrop spectrophotometer (Thermo Scientific). RNA quality was evaluated with an Agilent Technologies 2100 Bioanalyzer. cDNA was generated using the Retroscript kit (Ambion).

We designed PCR primers to sequence the coding region of the candidate genes from exon sequences obtained from the Ensembl Database. PCR was carried out using eye cDNA in a 24μl PCR reaction containing 1xPCR buffer (10 mM Tris-HCl pH 8.3, 50 mM KCl), 250 μM of dATP, dCTP, dGTP, and dTTP, 0.2 μM of the forward and the reverse primer, 1.5 mM MgCl_2_, and 0.6 U Taq polymerase. The following PCR program was used: 94°C for 90 sec, followed by 35 cycles of 94°C for 30 sec, 55°C for 45 sec, and 72°C for 45 sec, and a final extension of 72°C for 2 minutes. PCR products were electrophoresed on 1% agarose gels and visualized by ethidium bromide staining. DNA fragments were sequenced on an Applied Biosystems 3730XL (using a 50 cm array and POP7 polymer).

### Histological analysis

Mice were euthanized by carbon dioxide inhalation. Eyes were enucleated and fixed overnight in cold methanol:acetic acid:PBS (3:1:4). After paraffin embedding, eyes were cut into 4 μm sections, stained by H&E, and examined by light microscopy. For collection of brain and testes, whole body perfusion with PBS followed by Bouin’s fixative was performed immediately postmortem. Tissue specimens were collected and fixed in Bouin’s fixative overnight at room temperature. Tissues were dehydrated, embedded in paraffin and sectioned at 4 μm thickness. Testes and sagittal brain sections were stained with toluidine blue stain and H&E stain, respectively. For analysis of muscle dystrophy in *Fkrp*^*tvrm53*^ mice, segments of the spine or hindlimb were fixed and decalcified in Bouin’s for one week. Tissues were dehydrated, embedded in paraffin, cut into 4 μm sections and stained by H&E. Histologically stained sections were examined by light microscopy using a NanoZoomer slide scanner (Hamamatsu) at 20x resolution. Nuclear distribution in muscle fibers was quantified using Fiji. Following an initial Process>Smooth operation, H&E-stained images were separated by Image>Color>Colour Deconvolution into channels corresponding to nuclei (hematoxylin stain) and muscle fibers (eosin stain). Regions of interest (ROIs) bounding individual fibers were selected manually from the fiber channel using the Cell Magic Wand Tool. A macro was then used to list all instances in which a fiber ROI overlapped a nucleus identified from a binary mask of the thresholded nuclear channel. A second macro listed all instances of fibers containing a central nucleus, which was determined from a masked image where each ROI was eroded to exclude nuclei near its boundary. The ROI lists were processed and counted by copying unique records using Data>Advanced Filter in Excel. The number of fibers with one or more central nucleus was divided by the total number of fibers that had one or more nuclei to give the percentage of fibers with central nuclei. Independent determinations were analyzed statistically in Prism (GraphPad Software) using a two-tailed unpaired Student’s t-test with unequal variances.

### Immunohistochemistry (IHC)

The protocol for immunohistochemical assays was previously described [125]. Briefly, after carbon dioxide asphyxiation, eyes were enucleated and placed in cold methanol:acetic acid:PBS (3:1:4) or freshly prepared 4% paraformaldehyde (PFA) in PBS overnight at 4°C, as appropriate for the application. The eyes were embedded in paraffin and deparaffinized 6 μm sections were incubated with anti-rhodopsin (Millipore MAB5356, 1:200, or NeoMarkers MS-1233-R7, undiluted), anti-GFAP (DAKO Z0334, 1:200), anti-β-dystroglycan (Novocastra NCL-b-DG 1:100), anti-FKRP (Novus NBP1-74745, 1:200), anti-CTBP2 (BD Biosciences, 612044, 1:200), anti-collagen IV (Chemicon AB756P, 1:200), ezrin (Cell Signaling 3145, 1:200), anti-cone arrestin (Millipore AB15282, 1:200), or anti-α-laminin (Sigma L9393, 1:100). Antibody binding was revealed using species-specific Cy3- or Alexa Fluor488-conjugated anti-IgG (Jackson Immunoresearch and Life Technologies, 1:200) and visualized by fluorescence microscopy. For negative controls, the primary antibody was omitted.

### Western blotting (WB)

Mouse eye tissues were homogenized on ice in RIPA lysis buffer [1% NP40, 0.5% sodium deoxycholate, 0.1% SDS in phosphate buffered saline with Roche Complete Mini proteinase inhibitor (Sigma)] and centrifuged at 16,100 x g for 20 min. Protein lysates (40 μg/sample) were electrophoresed in a 10% Mini PROTEAN TGX gel (Bio-Rad) and transferred onto a nitrocellulose membrane using the TransBlot Turbo System (Bio-Rad). Membranes were pre-blocked in Blotto A solution (5% w/v milk powder in 0.05% v/v Tween in Tris-buffered saline [TBST]) for one hour at room temperature and incubated with primary antibody in Blotto A solution overnight at 4°C. Membranes were washed three times in TBST and incubated with peroxidase-conjugated secondary antibody for one hour at room temperature. Following three washes in TBST, immunobands were visualized by luminography using Clarity Western ECL Substrate (Bio-Rad). Antibodies used for WB analysis include: anti-Pde6α (Proteintech, 1:1000), anti-Rom1 (Proteintech, 1:1000), and anti-β-actin (Sigma, 1:2000).

### Ultrastructural analysis

Methods for preparation of retinal tissues for scanning and transmission EM have been previously described [146]. Briefly, for scanning analysis, eyes were immersed in 3% w/v glutaraldehyde, 1% w/v paraformaldehyde in cacodylate buffer for 2–4 hours at 4°C. RPE-free retinal specimens were osmicated, dehydrated, treated with hexamethyldisilazane and sputter coated. Photoreceptor OSs were examined by SEM at 20kV (Hitachi Technologies America, Parsippany, NJ). For transmission EM, eyes were immersed in cold 2.5% w/v glutaraldehyde, 2% w/v paraformaldehyde in 0.1 M phosphate buffer (pH 7.2) for 4 hours. Dissected retinas were post-fixed in osmium tetroxide, dehydrated and embedded in plastic resin. Ultrathin sections were stained with uranyl acetate and lead citrate and examined using a transmission electron microscope (JEOL JEM-1230).

## Supporting information

S1 Table TVRM mutants characterized as of September 1, 2016. (DOCX)

S1 Fig Protein structural models of predicted missense variants encoded by new TVRM alleles. Structural models available from SWISS-MODEL and MODBASE are displayed as rockets (α-helices) and planks (β-sheets) using Jmol (www.jmol.org/). The backbone positions of residues substituted in missense variants are colored red. Other features are colored yellow as indicated. (A) AIPL1, amino acid residues 12–326 of 328 total (SWISS-MODEL Q924K1, 1kt0A template). (B) RHO, residues 1–348 of 348 (SWISS-MODEL P15409, 3oaxA template). The retinylidene chromophore is highlighted in yellow. (C) CLCN2, residues 102–556 of 908, encompassing the transmembrane domain (SWISS-MODEL Q9R0A1, MODBASE 1otsA template). The chloride ion selectivity filter is highlighted in yellow. (D) FKRP, residues 324–472 of 494, corresponding to the LicD nucleotidyltransferase domain (SWISS-MODEL Q8CG64, 4e8iA template). A triad of active-site aspartic acid residues is highlighted in yellow.
(PDF)

S2 Fig Retinal degeneration in homozygous *Rho*^*Tvrm334*^ mice. (A) Retinal sections stained with H&E showed a progressive reduction in ONL thickness of homozygous *Rho*^*Tvrm334*^ mice at P14 (n = 4) and P21 (n = 4) compared to B6J control mice at P14 (n = 3). Retinal layers are labeled as in Fig 1B. *Bar*, 25 μm. (B) Immunostaining with anti-rhodopsin antibody (*green*) and DAPI (*blue*) indicated an increased mislocalization of RHO to cell soma in homozygous *Rho*^*Tvrm334*^ mice (n = 4) compared to B6J control mice (n = 4), both at P14. OS, outer segment. *Bar*, 20 μm.
(PDF)

S3 Fig Schematic diagram of the effect of the *Alms1*^*tvrm102*^ allele. The mutation is a splice donor mutation, which causes aberrant splicing of 120 basepairs into intron 6.
(PDF)

S4 Fig Hindlimb dystrophy in homozygous *Fkrp*^*tvrm53*^ mutant mice. Histological sections stained with H&E showed mislocalized nuclei in hindlimb muscle in homozygous *Fkrp*^*tvrm53*^ (*tvrm53*) (n = 4) compared to B6J (n = 3) mice at one year of age.
(PDF)
