# Supplementary material for: Mouse models of human ocular disease for translational research
Source: PLoS One. 2017 Aug 31;12(8):e0183837. doi: 10.1371/journal.pone.0183837 (PMC5578669; doi:10.1371/journal.pone.0183837)
Supplement: S1 Table — (DOCX) [file pone.0183837.s001.docx]

**S1 Table. TVRM mutants characterized as of September 1, 2016.**

| **Model** | **Mode^a^** | **Gene** | **Chr^b^** | **Mutation** | **Clinical Phenotype** | **Reference** |
| --- | --- | --- | --- | --- | --- | --- |
| *tvrm267* | AR | *Adamtsl4* | 3 | p.Gln609* | RPE defects | [[147](#_ENREF_147)] |
| *tvrm119* | AR | *Aipl1* | 11 | p.Val33_Ile92del | Early rapid retinal degeneration | this report |
| *tvrm127* | AR | *Aipl1* | 11 | p.Cys89Phe | Early rapid retinal degeneration | this report |
| *tvrm102* | AR | *Alms1* | 6 | c.1080+2T>C | Retinal degeneration, weight gain | this report |
| *nmf240* | AR | *Clcn2* | 16 | p.Glu355* | Early rapid retinal degeneration | [[46](#_ENREF_46)] |
| *nmf289* | AR | *Clcn2* | 16 | p.Gly482Val | Early rapid retinal degeneration | this report |
| *tvrm65* | AR | *Crx* | 7 | p.Leu277* | Early rapid retinal degeneration | [[24](#_ENREF_24)] |
| *tvrm5* | AR | *Ctnna1* | 18 | p.Leu436Pro | RPE defects | [[148](#_ENREF_148)] |
| *tvrm53* | AR | *Fkrp* | 7 | p.Ile356Thr | Vitreal fibroplasia | this report |
| *tvrm207* | AR | *Grk1* | 8 | p.Leu363Pro | Retinal degeneration | [[60](#_ENREF_60)] |
| *tvrm84* | AR | *Grm1* | 10 | p.Ile536Leu | Attenuated ERG | [[60](#_ENREF_60)] |
| *tvrm32* | AR | *Hps1^c^* | 19 | unknown | Pigmentation defect | unpublished |
| *nmf223* | AR | *Lama1* | 17 | p.Tyr265Cys | Vitreal fibroplasia, vascular abnormalities | [[126](#_ENREF_126)] |
| *tvrm257* | AR | *Lrit3* | 3 | p.Leu134Pro | No b-wave | [[60](#_ENREF_60)] |
| *tvrm111b* | AR | *Lrp5* | 19 | c.4724_4725insG | Reduced ERG b-wave | unpublished |
| *nmf12* | AR | *Mertk* | 2 | p.His716Arg | Late onset slow degeneration | [[149](#_ENREF_149)] |
| *tvrm89* | AR | *Myo6* | 9 | p.Leu480Pro | Attenuated ERG | [[150](#_ENREF_150)] |
| *tvrm113* | AR | *Nmnat1* | 4 | p.Asp243Gly | Early rapid retinal degeneration | [[61](#_ENREF_61)] |
| *nmf192* | AR | *Nphp4* | 4 | p.Leu104* | Early rapid retinal degeneration | [[151](#_ENREF_151)] |
| *nmf282* | AR | *Pde6a* | 18 | p.Val685Met | Early rapid retinal degeneration | [[152](#_ENREF_152)] |
| *nmf363* | AR | *Pde6a* | 18 | p.Asp670Gly | Early rapid retinal degeneration | [[152](#_ENREF_152)] |
| *tvrm58* | AR | *Pde6a^c^* | 18 | p.Leu638Pro | Early rapid retinal degeneration | [[25](#_ENREF_25)] |
| *nmf449* | AR | *Pde6b^c^* | 5 | unknown | Early rapid retinal degeneration | unpublished |
| *nmf5a* | AR | *Pfdn5* | 15 | p.Leu110Arg | Early rapid retinal degeneration | [[153](#_ENREF_153)] |
| *Tvrm1* | AD | *Rho* | 6 | p.Tyr102His | Light inducible retinal degeneration | [[42](#_ENREF_42)] |
| *Tvrm4* | AD | *Rho* | 6 | p.Ile307Asn | Light inducible retinal degeneration | [[42](#_ENREF_42)] |
| *Tvrm144* | AD | *Rho* | 6 | p.Trp35Arg | Light inducible retinal degeneration | [[24](#_ENREF_24)],[[25](#_ENREF_25)] |
| *Tvrm334* | AD | *Rho* | 6 | p.Tyr178Cys | Pan-retinal degeneration | this report |
| *tvrm64* | AR | *Rp1* | 1 | p.Arg522* | Juvenile onset retinal degeneration | [[24](#_ENREF_24)] |
| *tvrm148* | AR | *Rpe65* | 3 | p.Phe229Ser | Late onset retinal degeneration | [[24](#_ENREF_24)] |
| *nmf247* | AR | *Rpgrip1* | 14 | c.683-1A>T | Early rapid retinal degeneration | [[33](#_ENREF_33)] |
| *tvrm111* | AR | *Rpgrip1* | 14 | c.813+1G>A | Retinal degeneration | this report |
| *tvrm27* | AR | *Trpm1* | 7 | p.Ala1068Thr | No b-wave | [[137](#_ENREF_137)] |
| *tvrm124* | AR | *Tulp1^c^* | 17 | c.181+2T>C | Early rapid retinal degeneration | [[25](#_ENREF_25)] |

^a^Mode of inheritance. AR, autosomal recessive, AD, autosomal dominant. ^b^Chr, chromosome.  ^c^Established by complementation testing.

147. Collin GB, Hubmacher D, Charette JR, Hicks WL, Stone L, Yu M, et al. (2015) Disruption of murine *Adamtsl4* results in zonular fiber detachment from the lens and in retinal pigment epithelium dedifferentiation. Hum Mol Genet 24: 6958-6974.

148. Saksens NT, Krebs MP, Schoenmaker-Koller FE, Hicks W, Yu M, Shi L, et al. (2016) Mutations in *CTNNA1* cause butterfly-shaped pigment dystrophy and perturbed retinal pigment epithelium integrity. Nat Genet 48: 144-151.

149. Maddox DM, Hicks WL, Vollrath D, LaVail MM, Naggert JK, Nishina PM (2011) An ENU-induced mutation in the *Mertk* gene (*Mertk^nmf12^*) leads to a slow form of retinal degeneration. Invest Ophthalmol Vis Sci 52: 4703-4709.

150. Samuels IS, Bell BA, Sturgill-Short G, Ebke LA, Rayborn M, Shi L, et al. (2013) Myosin 6 is required for iris development and normal function of the outer retina. Invest Ophthalmol Vis Sci 54: 7223-7233.

151. Won J, Marin de Evsikova C, Smith RS, Hicks WL, Edwards MM, Longo-Guess C, et al. (2011) NPHP4 is necessary for normal photoreceptor ribbon synapse maintenance and outer segment formation, and for sperm development. Hum Mol Genet 20: 482-496.

152. Sakamoto K, McCluskey M, Wensel TG, Naggert JK, Nishina PM (2009) New mouse models for recessive retinitis pigmentosa caused by mutations in the *Pde6a* gene. Hum Mol Genet 18: 178-192.

153. Lee Y, Smith RS, Jordan W, King BL, Won J, Valpuesta JM, et al. (2011) Prefoldin 5 is required for normal sensory and neuronal development in a murine model. J BiolChem 286: 726-736.
