# Supplementary figures and images for: Mouse models of human ocular disease for translational research
Source: PLoS One. 2017 Aug 31;12(8):e0183837. doi: 10.1371/journal.pone.0183837 (PMC5578669; doi:10.1371/journal.pone.0183837)

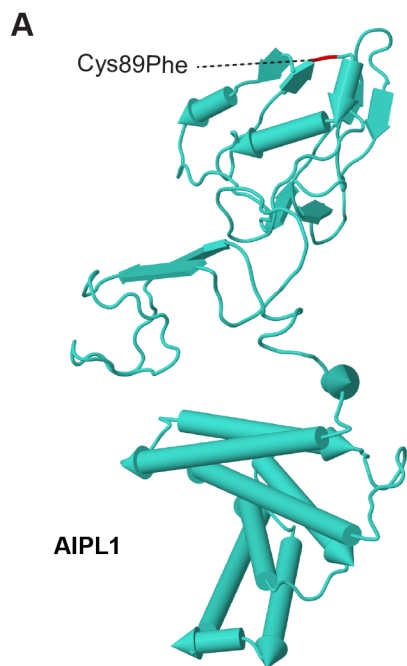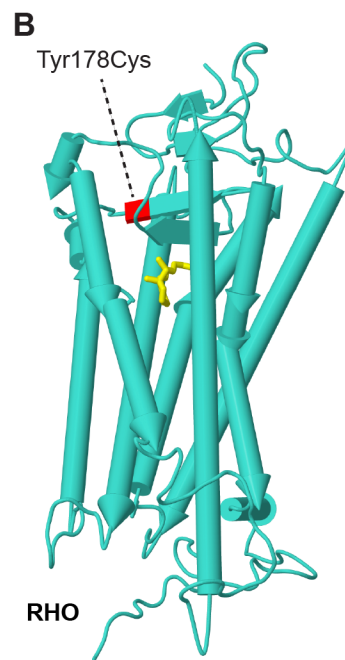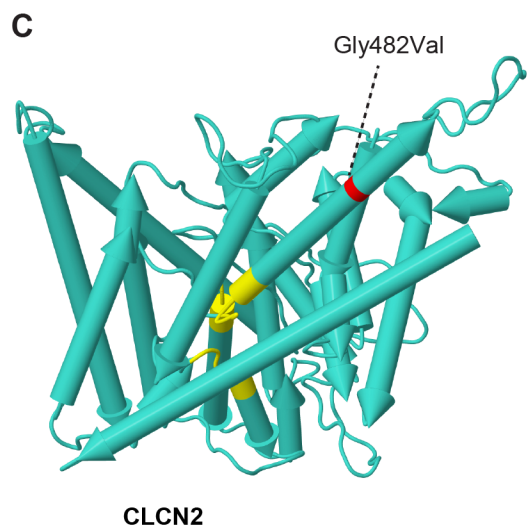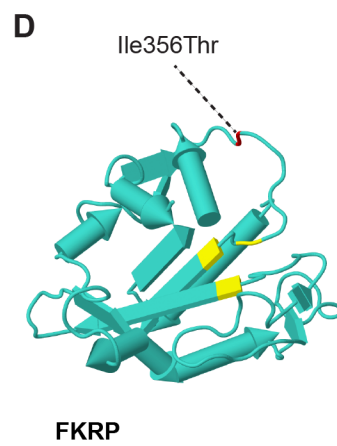

Supplement: S1 Fig — Structural models available from SWISS-MODEL and MODBASE are displayed as rockets (α-helices) and planks (β-sheets) using Jmol (www.jmol.org/). The backbone positions of residues substituted in missense variants are colored red. Other features are colored yellow as indicated. (A) AIPL1, amino acid residues 12–326 of 328 total (SWISS-MODEL Q924K1, 1kt0A template). (B) RHO, residues 1–348 of 348 (SWISS-MODEL P15409, 3oaxA template). The retinylidene chromophore is highlighted in yellow. (C) CLCN2, residues 102–556 of 908, encompassing the transmembrane domain (SWISS-MODEL Q9R0A1, MODBASE 1otsA template). The chloride ion selectivity filter is highlighted in yellow. (D) FKRP, residues 324–472 of 494, corresponding to the LicD nucleotidyltransferase domain (SWISS-MODEL Q8CG64, 4e8iA template). A triad of active-site aspartic acid residues is highlighted in yellow. (PDF) [file pone.0183837.s002.pdf]

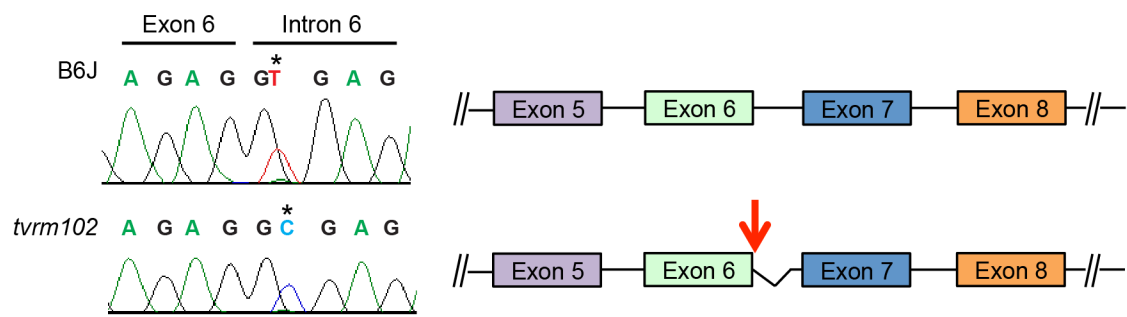

Supplement: S2 Fig — (A) Retinal sections stained with H&E showed a progressive reduction in ONL thickness of homozygous RhoTvrm334 mice at P14 (n = 4) and P21 (n = 4) compared to B6J control mice at P14 (n = 3). Retinal layers are labeled as in Fig 1B. Bar, 25 μm. (B) Immunostaining with anti-rhodopsin antibody (green) and DAPI (blue) indicated an increased mislocalization of RHO to cell soma in homozygous RhoTvrm334 mice (n = 4) compared to B6J control mice (n = 4), both at P14. OS, outer segment. Bar, 20 μm. (PDF) [file pone.0183837.s003.pdf]

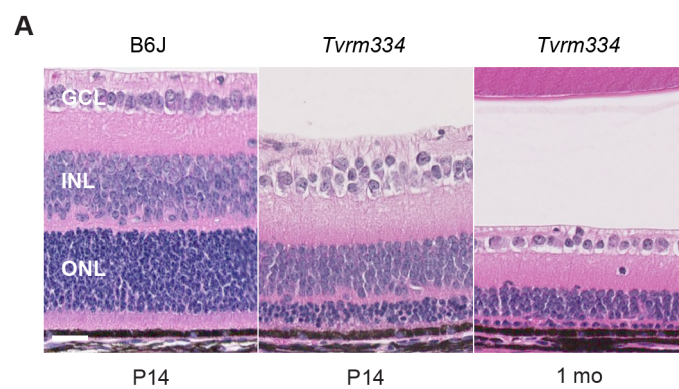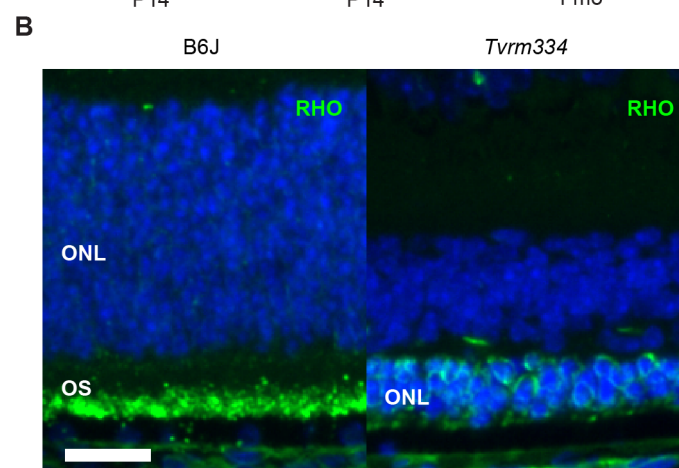

Supplement: S3 Fig — The mutation is a splice donor mutation, which causes aberrant splicing of 120 basepairs into intron 6. (PDF) [file pone.0183837.s004.pdf]

B6J

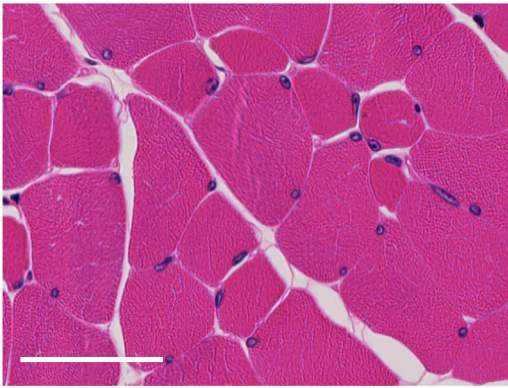

*tvrm53*

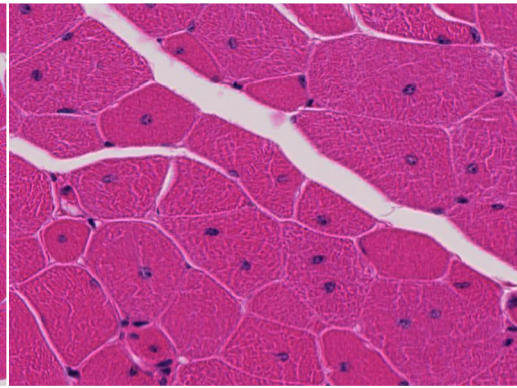

Supplement: S4 Fig — Histological sections stained with H&E showed mislocalized nuclei in hindlimb muscle in homozygous Fkrptvrm53 (tvrm53) (n = 4) compared to B6J (n = 3) mice at one year of age. (PDF) [file pone.0183837.s005.pdf]
